# Uncovering the shell game with barcodes: diversity of meiofaunal Caecidae snails (Truncatelloidea, Caenogastropoda) from Central America

**DOI:** 10.3897/zookeys.968.52986

**Published:** 2020-09-16

**Authors:** Christina Egger, Timea P. Neusser, Jon Norenburg, Francesca Leasi, Barbara Buge, Angelo Vannozzi, Regina L. Cunha, Cymon J. Cox, Katharina M. Jörger

**Affiliations:** 1 SNSB-Zoologische Staatssammlung München, Münchhausenstr. 21, 81247 Munich, Germany SNSB-Zoologische Staatssammlung München Munich Germany; 2 CCMAR, Campus de Gambelas, Universidade do Algarve, 8005-139 Faro, Portugal Universidade do Algarve Faro Portugal; 3 LMU Munich, Biocenter, Dept. II, Großhaderner Str. 2, 82152 Planegg-Martinsried, Germany LMU Munich Munich Germany; 4 Department of Invertebrate Zoology, National Museum of Natural History, Smithsonian Institution, Washington, DC 20560, USA National Museum of Natural History Washington, DC United States of America; 5 Department of Biology, Geology and Environmental Science. University of Tennessee at Chattanooga. 615 McCallie Ave. Chattanooga, TN 37403, USA University of Tennessee at Chattanooga Chattanooga United States of America; 6 Muséum national d’Histoire naturelle, 55 Rue Buffon, 75231 Paris, France Muséum national d’Histoire naturelle Paris France; 7 Independent researcher, Via M.L. Longo 8, Rome, Italy Unaffiliated Rome Italy

**Keywords:** DNA taxonomy, marine biodiversity, meiofauna, molecular species delineation, Mollusca

## Abstract

Caecidae is a species-rich family of microsnails with a worldwide distribution. Typical for many groups of gastropods, caecid taxonomy is largely based on overt shell characters. However, identification of species using shell characteristics is problematic due to their rather uniform, tubular shells, the presence of different growth stages, and a high degree of intraspecific variability. In the present study, a first integrative approach to caecid taxonomy is provided using light-microscopic investigation with microsculptural analyses and multi-marker barcoding, in conjunction with molecular species delineation analyses (ABGD, haplotype networks, GMYC, and bPTP). In total 132 specimens of *Caecum* and *Meioceras* collected during several sampling trips to Central America were analyzed and delineated into a minimum of 19 species to discuss putative synonyms, and supplement the original descriptions. Molecular phylogenetic analyses suggest *Meioceras nitidum* and *M. cubitatum* should be reclassified as *Caecum*, and the genus *Meioceras* might present a junior synonym of *Caecum*. Meiofaunal caecids morphologically resembling *C. glabrum* from the Northeast Atlantic are a complex of cryptic species with independent evolutionary origins, likely associated with multiple habitat shifts to the mesopsammic environment. *Caecum invisibile* Egger & Jörger, **sp. nov.** is formally described based on molecular diagnostic characters. This first integrative approach towards the taxonomy of Caecidae increases the known diversity, reveals the need for a reclassification of the genus *Caecum* and serves as a starting point for a barcoding library of the family, thereby enabling further reliable identifications of these taxonomically challenging microsnails in future studies.

## Introduction

In the past fifteen years molecular barcoding and molecular species delineation have revolutionized the assessment of species diversity and traditional taxonomy, allowing for fast and reproducible species identification and delimitation, and adding to objectivity and reliability in species diagnoses (Leasi et al. 2013; Fontaneto et al. 2015; Scarpa et al. 2016; Martínez-Arce et al. 2020). Molecular data enables testing for morphologically cryptic species as well as phenotypic plasticity, and the evaluation of intra- versus inter-specific variability (Jörger et al. 2012; Leasi et al. 2013, 2016). Given the number of described species and 250 years of taxonomic practice that delimit species based largely on distinct morphologies, it is unsurprising that despite the success of modern molecular approaches, many clades of Metazoa have yet to have their morphological classification tested against molecular markers.

Traditionally, the taxonomy of Gastropoda, one of the most species-rich and better-known clades of invertebrates in the marine environment, is largely based on shell characteristics (Bouchet and Strong 2010). However, this approach is generally problematic as several studies have revealed species exhibiting phenotypic plasticity in shell form due to environmental factors or predation (Trussell 2000; Weigand et al. 2011), and uncovered cryptic species with the aid of molecular data (Haase et al. 2007; Puillandre et al. 2010; Jörger et al. 2012). Consequently, these studies question evolutionary hypotheses based on species delimited by shell characteristics alone and point to the need for an integrative approach using both molecular and morphological data in future research.

**Figure 1.**
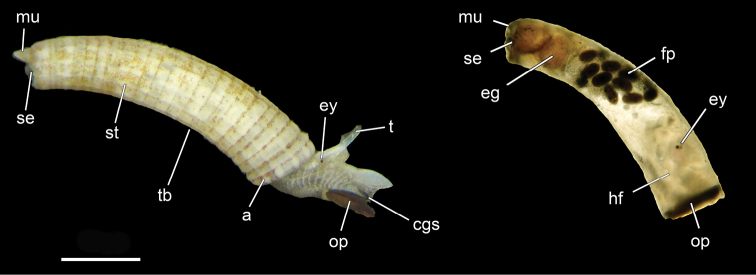
Morphology of *Caecum* (Caecidae) including important shell features used for morphological species identification. USNM 1618850 *Caecum imbricatum* – specimens to the left. Abbreviations: a, aperture; cgs, ciliated gliding sole; eg, egg; ey, eye; fp, fecal pellets; hf, retracted head and foot; mu, mucro; op, operculum; se, septum; st, shell structure (ornamentation); t, tentacle; tb, tube. Scale bar: 500 µm.

Members of the family Caecidae Gray, 1850 can be found in different marine habitats (e.g., among algae or corals) including the marine mesopsammon (i.e., the aqueous interstitial pore spaces of marine sediments). As adults they have uncoiled tubular shells that are likely an adaptation to their infaunal lifestyle (Swedmark 1968). In early descriptions zoologists associated Caecidae snails with tusk-shells (nowadays known as scaphopod molluscs) (see e.g., Montague 1803) or classified them among annelid tube worms (Brown 1827; see Pizzini et al. 2013 for a classificatory history). Even after Caecidae were settled among gastropods (Clark 1849), with current phylogenetic hypotheses placing them among caenogastropod Truncatelloidea (Criscione and Ponder 2013), their unusual tubular shells still posed challenge to taxonomists. Caecid larval shells (protoconch) are usually planspirally coiled with two whorls (Bandel 1996) and closely resemble related gastropod veliger shells. After settlement of the larvae the adult shell (teleoconch) is formed through differing degrees of uncoiling, with the protoconch either remaining attached (*Parastrophia* de Folin, 1869, *Ctiloceras* R. B. Watson, 1866, *Enigmerces* Iredale & Laseron, 1957, *Jayella* Iredale & Laseron, 1957, *Ponderoceras* Bandel, 1996, *Strebloceras* Carpenter, 1859) or being shed (*Caecum* J. Fleming, 1813, *Meioceras* Carpenter, 1859, *Pizzinia* Vannozzi, 2017, and *Mauroceras* Vannozzi, 2019). In the latter case, the growing teleoconch is closed by a septum (Bandel 1996). The snails continue to shed part of the teleoconch until the fully developed adult shell is formed (Draper 1974). The number of repetitions of shedding likely is variable between species, but unknown for the majority of caecids (Pizzini et al. 1998). This complex shell ontogeny results in highly variable shell morphologies during ontogeny (with a minimum of three different shell morphologies: the larval shell-form, the juvenile shell form(s) and the adult shell form), which hampers species identification and delineation based on single shells if no comparative data is available for the entire morpho-series (i.e., all developmental stages). Moreover, the tubular shells have few taxonomic characters, thus the current taxonomy is largely based on conchological characters such as size, shell shape, ornamentation, construction of the aperture, septum and mucro (i.e., an evagination of the septum, see Fig. 1 for terminology) (Lightfoot 1992a, b, 1993a, b; Pizzini and Raines 2011; Pizzini et al. 2013; Vannozzi et al. 2015; Vannozzi 2017). However, these characters can change for an individual during its lifetime, for instance, young specimens can be entirely smooth and express shell ornamentation only later during maturation, and also shell shape may change as they continue to add shell material at their aperture (i.e., shell opening, see Fig. 1) (Draper 1974; Pizzini 1998b; Lima et al. 2013). Additional difficulties arise in determining whether the septum and mucro are temporary or final (Pizzini et al. 1998).

While the phylogenetic position of the family among truncatelloid gastropods is supported by molecular and morphological data, the taxonomy within the family still is based largely only on shell morphology alone. Indeed, anatomical data is scarce (e.g., Götze 1938; Draper 1974) and thought to offer few diagnostic characters, while molecular barcoding approaches are lacking entirely. Currently, the family Caecidae contains approx. 260 described species in ten genera (MolluscaBase 2019). Most genera (i.e., *Strebloceras*, *Ctiloceras*, *Jayella*, *Enigmerces*, *Ponderoceras*, *Pizzinia*, and *Mauroceras*) are species-poor and limited in distribution to the Indo-Pacific (Iredale and Laseron 1957; Bandel 1996; Pizzini et al. 2013; Vannozzi 2016, 2017). Only *Caecum*, currently with 210 valid species (according to MolluscaBase 2019), shows a circumglobal distribution in temperate and tropical zones. Their abundance is particularly high in tropical waters such as the Indo-Pacific and Central America (Vokes 1983; De Jong and Coomans 1988; Lightfoot 1992a, b, 1993a, b; Díaz Merlano and Puyana Hegedus 1994; Pizzini 1998a; Pizzini and Bonfitto 2008; Discover Life 2020). *Meioceras*, which differs from *Caecum* in the general shape of the shell (i.e., with the widest part towards the middle of the shell), was erected by Carpenter (1858–1859) due to the slightly coiled shape of their juveniles. The genus was recently split into Indo-West Pacific *Mauroceras* and Western Atlantic *Meioceras* (Vannozzi 2019). While recent taxonomic works have described the caecid fauna in the Indo-Pacific based on microsculptural investigations of the shell (Pizzini et al. 2013; Vannozzi 2017, 2019), knowledge of caecid diversity in Central American waters is still limited to light-microscopic identification of shells for a large majority of described species.

In this study we present data on caecid diversity based on several recent collecting trips to Central America. We identified the collected Caecidae specimens based on traditional taxonomy and used additional microsculptural observations and molecular barcodes to reliably assign different growth stages to taxa. We applied an integrative experimental approach including multi-marker barcoding and molecular species delineation analyses to test our morphology-based taxonomy, and to identify putative cryptic species.

## Materials and methods

**Figure 2.**
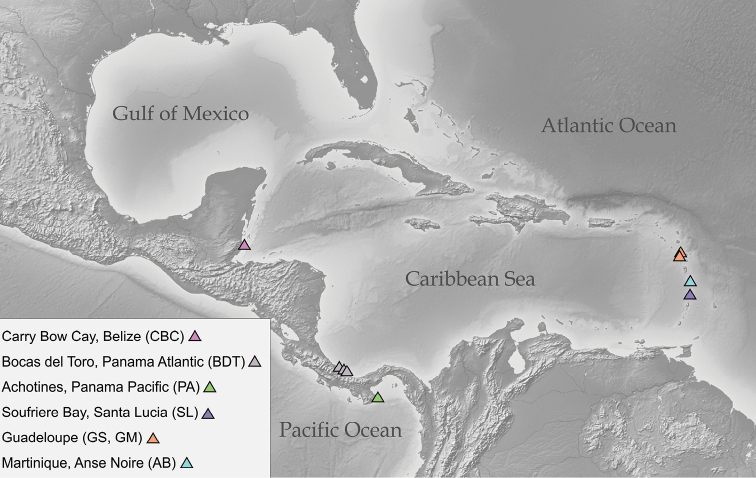
Map of color-coded sampling sites (triangles) for Caecidae in Central American waters.

We collected and microscopically investigated a total of 132 individuals of meiofaunal caecid snails from five different sites in tropical Central America. Of 132 specimens, 67 were selected for further analyses (see Fig. 2 for sampling sites and Tables 1, 2 for details on material and sampling sites). Specimens were extracted from samples of coarse subtidal sands by resting them in buckets for at least 1–2 days to deplete oxygen and accumulate the meiofauna in the surface layer. The surface layer was skimmed off, and the snails extracted by a decantation technique after anesthetization with MgCl_2_-seawater solution using a sieve with a mesh size of 100 µm (Jörger et al. 2014). All specimens were documented alive and grouped into preliminary morphotypes based on light microscopic (LM) examination of shell characters in the field and fixed in 75–96% ethanol. Specimens provided by the Muséum national d’Histoire naturelle (**MNHN**) Paris had previously been removed from their shells in the field by the use of a microwave oven (Galindo et al. 2014), this method is advantageous and recommended over the destructive sampling described below, applied in the beginning of our survey.

### Shell characteristics and microsculptural analyses

We documented the main taxonomic characters of the tubular shells (Fig. 1), such as the morphology of aperture, septum, and mucro, and measured size and diameter of the shells. Initial species identification in the field was carefully revised in the laboratory, and specimens were assigned to species according to these shell characteristics. The microsculpture of the shell of one representative of each putative morphospecies was investigated via scanning electron microscopy (SEM), whenever a voucher was available (Table 1).

**Table 1.** List of investigated Caecidae specimens, museums numbers (**ZSM**: SNSB-Bavarian State Collection, **MNHN**: Muséum National d’Histoire Naturelle, **USMN**: Smithsonian Institution), and **NCBI** GenBank accession numbers of sequenced genes and the type of voucher of the material of Caecidae analyzed in the present study. An asterisk (*) marks individuals used for SEM scans.

Species	Field-code	Locality code	Specimen catalog number	Voucher	GenBank number		
					COI	16S rRNA	28S rRNA
*Caecum imbricatum*	CBC_26	CBC3	USNM 1618850	DNA	MT727051	MT704281	
*Caecum imbricatum*	BDT_04	BRS101	USNM 1618852	DNA		MT704261	
*Caecum imbricatum*	BDT_07	BRS103	USNM 1618854	DNA	MT727047		
*Caecum imbricatum*	BDT_08	BRS103	USNM 1618855	DNA	MT727048		
*Caecum striatum*	CBC_8	CBC24	USNM 1618845	DNA	MT727061	MT704275	
*Caecum invisibile* sp. nov.	CBC_1bB	CBC1b	ZSM-Mol-20200109	DNA, paratype	MT727054	MT704267	MT731696
*Caecum invisibile* sp. nov.	CBC_1bC	CBC1b	ZSM-Mol-20100320	DNA*, holotype	MT727055	MT704268	MT731697
*Caecum invisibile* sp. nov.	CBC_3a	CBC1b	USNM 1618839	DNA	MT727056	MT704269	MT731698
*Caecum invisibile* sp. nov.	CBC_3c	CBC1b	USNM 1618840	DNA	MT727057	MT704270	MT731699
*Caecum invisibile* sp. nov.	CBC_3d	CBC1b	USNM 1618841	DNA	MT727058	MT704271	MT731700
*Caecum invisibile* sp. nov.	CBC_3e	CBC1b	USNM 1618842	DNA	MT727059	MT704272	MT731701
*Caecum invisibile* sp. nov.	CBC_3f	CBC1b	USNM 1618843	DNA	MT727060	MT704273	MT731702
*Caecum invisibile* sp. nov.	CBC_13a	CBC1b	USNM 1618846	DNA	MT727062	MT704276	MT731704
*Caecum invisibile* sp. nov.	CBC_13b	CBC1b	USNM 1618847	DNA	MT727063	MT704277	MT731705
*Caecum invisibile* sp. nov.	CBC_13c	CBC1b	USNM 1618848	DNA	MT727064	MT704278	MT731706
*Caecum invisibile* sp. nov.	CBC_13d	CBC1b	USNM 1618849	DNA	MT727065	MT704279	MT731707
*Caecum invisibile* sp. nov.	BDT_20	BRS104	USNM 1618856	DNA	MT727049	MT704264	MT731689
*Caecum invisibile* sp. nov.	BDT_48	BRS200	USNM 1618859	DNA	MT727052		MT731694
*Caecum regulare*	BDT_22	BRS108	USNM 1618883	DNA	MT727050		MT731690
*Caecum regulare*	CBC_22B	CBC22	ZSM-Mol-20100321	DNA*		MT704280	MT731708
*Caecum donmoorei*	BDT_23	BRS108	USNM 1618857	DNA		MT704265	MT731691
*Caecum donmoorei*	BDT_25	BRS108	USNM 1618858	DNA		MT704266	MT731692
*Caecum donmoorei*	CBC_6	CBC1b	USNM 1618844	DNA		MT704274	MT731703
MOTU I	BDT_17		ZSM-Mol-20200039	DNA*		MT704263	MT731688
MOTU II	BDT_06	BRS101	USNM 1618853	DNA	MT727046	MT704262	MT731687
MOTU II	BDT_46	BRS110	USNM 1618852	DNA	MT727051		MT731693
MOTU II	BDT_49	BRS200	USNM 1618860	DNA	MT727053		MT731695
Caecum cf. corrugulatum	PA_C04	PA14	USNM 1618861	DNA	MT727069		MT731722
*Caecum heptagonum*	PA_28A	PA23a	ZSM-Mol-20200030	DNA*		MT704283	MT731717
*Caecum heptagonum*	PA_G10	PA23a	USNM 1618866	DNA		MT704291	MT731726
Caecum cf. teres	PA_E10	PA23a	USNM 1618865	DNA	MT727070	MT704289	MT731724
Caecum cf. teres	PA_30B	PA23a	ZSM-Mol-20200033	DNA*		MT704284	MT731718
Caecum cf. teres	PA_30G	PA23a	ZSM-Mol-20200037	DNA*		MT704286	MT731720
Caecum cf. strangulatum	PA_G12	PA23a	USNM 1618884	DNA	MT727072		MT731727
Caecum cf. strangulatum	PA_A07	PA14	USNM 1618864	DNA	MT727068	MT704287	MT731721
Caecum cf. semilaeve	PA_11B	PA14	ZSM-Mol-20200028	DNA*		MT704282	MT731716
Caecum cf. semilaeve	PA_30C	PA23a	ZSM-Mol-20200034	DNA*		MT704285	MT731719
*Caecum* sp.	PA_H05	PA15	USNM 1618862	DNA		MT704292	MT731728
*Caecum* sp.	PA_E06	PA14	USNM 1618885	DNA		MT704288	MT731723
*Caecum* sp.	PA_F06	PA14	USNM 1618886	DNA	MT727071	MT704290	MT731725
*Caecum* sp.	PA_H06	PA14	USNM 1618863	DNA	MT727073	MT704293	
*Caecum pulchellum*	SL_01	SL1	ZSM-Mol-20090485	DNA*	MT727074	MT704300	MT731729
*Caecum cooperi*	Gu12_20	GS32	MNHN-IM-2019-32	DNA, shell*		MT704297	MT731713
Caecum cf. clathratum	Gu12_06	GM01	MNHN-IM-2019-17	DNA, shell*		MT704294	MT731710
*Caecum debile*	Gu12_15	GS32	MNHN-IM-2019-27a	DNA, shell*		MT704295	MT731711
*Caecum debile*	Gu12_16	GS32	MNHN-IM-2019-27b	DNA, shell		MT704296	MT731712
*Meioceras nitidum*	Ma16_01	AB102	MNHN-IM-2013-2087a	DNA, shell*		MT704298	MT731714
*Meioceras nitidum*	Ma16_02	AB102	MNHN-IM-2013-2087b	DNA, shell		MT704299	MT731715
*Meioceras cubitatum*	CBC_44	CBC15	USNM 1618851	DNA	MT727067		MT731709

Microscopic debris on the shell was manually removed using an eyelash, and the shell rinsed in 96% ethanol. Specimens were dried by evaporation of the ethanol and transferred onto SEM stubs covered with self-adhesive carbon stickers. We used a sputter coater Polaron SC510 to coat the samples with gold in argon atmosphere. The shells were analyzed with a LEO 1430 VP SEM at a voltage of 15 kV.

All light microscopic images and SEM-micrographs are available through FigShare (https://figshare.com/projects/Central_American_Caecidae/84929).

**Table 2.** Details on sampling localities and habitat of the investigated specimens.

Locality code	Region	Station	Latitude, Longitude	Depth	Date	Habitat
CBC1b	Carrie Bow Cay, Belize	House reef	16.8015, -88.0790	10 m	14/01/2010	open plain
CBC3	Carrie Bow Cay, Belize	House reef	16.8037, -88.0769	31 m	15/01/2010	trough inside ridge
CBC15	Carrie Bow Cay, Belize	House reef	16.8021, -88.0768	31 m	22/01/2010	trough inside ridge
CBC22	Carrie Bow Cay, Belize	Curlew Reef	16.7911, -88.0761	15 m	24/01/2010	protected sand in patches
CBC24	Carrie Bow Cay, Belize	House reef	16.8024, -88.0776	19 m	25/01/2010	small sand patches on ridge
BRS101	Bocas del Toro, Panama Atlantic	South of Punta Cauro	9.3609, -82.3467	3 m	08/06/2010	small sandy patches, silty, medium coarse sand
BRS103	Bocas del Toro, Panama Atlantic	Solarte Garden	9.3222, -82.2215	4.5 m	09/06/2010	exposed, sandy patches, silty, fine
BRS104	Bocas del Toro, Panama Atlantic	Wild Cane Rock	9.3503, -82.1723	14 m	10/06/2010	deep, sand plain, long ripples, medium coarse sand
BRS108	Bocas del Toro, Panama Atlantic	Near Tiger Rock	9.2141, -81.9318	8.5 m	10/06/2010	n/a
BRS110	Bocas del Toro, Panama Atlantic	Wild Cane Reef	9.3507, -82.1724	15 m	12/06/2010	sand plain, medium coarse sand
BRS200	Bocas del Toro, Panama Atlantic	Wild Cane Reef	9.3507, -82.1724	3 m	12/06/2010	coarse sand 200 µm
PA4	Achotines, Panama Pacific	Achotines Bay	7.4145, -80.1765	2–4 m	25/02/2016	sand pits between corals, coarse sand
PA12	Achotines, Panama Pacific	Back of Achotines Laboratory	7.4119, -80.1735	intertidal-subtidal	28/02/2016	tide pools, wave action, scoarse sand
PA14	Achotines, Panama Pacific	Isla Iguana south	7.6207, -80.0013	12 m	29/02/2016	sandy plain around rocks, lots of organic matter, coarse to fine
PA15	Achotines, Panama Pacific	Isla Iguana west	7.6301, -80.0022	11–16 m	29/02/2016	slope with coral rubble, coarse to fine
PA23a	Achotines, Panama Pacific	Isla Iguana north	7.6349, -79.9968	10 m	06/03/2016	sand plain, partially with organic matter, gravel and coarse
PA23b	Achotines, Panama Pacific	Isla Iguana north	7.6346, -79.9965	10 m	06/03/2016	patches next to rocky coral, gravel, course
SL1	Santa Lucia	Soufriere Bay	13.8494, -61.0675	8–9m	19/02/2009	
GS32	Guadeloupe	west Fajou	16.3558, -61.5965	2	24/05/2012	lagoon terrace with sandy bottom
GM01	Guadeloupe	small marine dead end	16.2235, -61.5305	1	02/05/2012	
AB102	Martinique	Anse Noire	14.5283, -61.0883	6	06/09/2016	

### DNA extraction, amplification, and sequencing

DNA was extracted from 121 of the 132 investigated specimens. The sputter-coated individuals previously investigated by SEM were crushed mechanically using pestles (Bergmeier et al. 2016); specimens investigated only by LM were also crushed if tissue was not already separated. Subsequently, DNA was extracted by the procedure of Knebelsberger and Stöger (2012) combining lysis with 2-mercaptoethanol in CTAB buffer, chloroform-isoamyl precipitation, and recovery using columns with silica-membrane (Nucleo Spin, Macherey-Nagel GmbH & Co. KG, Düren, Germany). The DNA was eluted twice with 25 µl aliquots of pre-heated elution buffer to gain high yield. DNA of specimens deposited to the USMN-Smithsonian Institution were extracted in the Laboratories for Analytical Biology, SI using the standard protocols of the Autogen Prep 956 Extractor (eluting with 100 µl Autogen R9 buffer). Three different markers were partially amplified by PCR: mitochondrial cytochrome oxidase subunit I (COI) and 16S rRNA gene, and nuclear 28S rRNA gene, using the standard PCR primers for gastropods (see Klussmann-Kolb et al. 2008). We either used the Phire polymerase (Thermo Fisher Scientific Inc., Waltham, USA) with the following protocol for PCRs on 16S/ COI resp. 28S at the LMU: 98 °C 1 min (98 °C 30 sec; 46–50 °C 20 sec; 72 °C 20 sec) × 36–38 cycles 72 °C 1 min resp.98 °C 30 sec (98 °C 15 sec; 55–60 °C 5 sec; 72 °C 20 sec) × 35 cycles 72 °C 1 min or the KlenTaq polymerase (AB Peptides, Inc.) with the following program for sequences generated at the SI: 95 °C 3 min, (95 °C 30–45 sec; 48–52 °C 30–45 sec; 72 °C 45–90 sec) × 35–40, 72 °C 7 min. PCR products were either cleaned using a spin column purification kit (Zymo Research, Irvine, California, USA) or were purified with QIAquick (Qiagen Inc.). Samples at the LMU were cycle sequenced on an ABI 3730 48 capillary sequencer (Applied Biosystems, Foster City, USA) using Big Dye 3.1 (Thermo Fisher Scientific Inc.) at the sequencing service of the Ludwig-Maximilians-Universität (LMU) Biocenter, Munich, Germany. At the SI, cycle sequencing was also conducted with BigDye chemistry (PerkinElmer) and standard cycles (4 min denaturation at 96 °C, followed by 25 cycles of 10 sec at 96 °C, 5 sec at 50 °C and 4 min at 60 °C), and sequenced on an ABI 3730xl 96-well capillary sequencer. In total, 34%, 43% and 50% of the partial COI, 16S rRNA, and 28S rRNA gene sequences, respectively, were successfully amplified and sequenced. All sequences were edited in Geneious Prime (vers. 11.02011, Biomatters, Ltd., Auckland, New Zealand). Primer sequences were removed and base calls checked for misreads against their chromatogram. The sequences were then compared to sequences in the public database NCBI GenBank (http://ncbi.nlm.nih.gov/genbank) by using the BLAST online web service to check for putative contamination. In total 29 COI, 40 16S rRNA and 43 28S rRNA gene sequences were deposited in NCBI GenBank (see Table 1 for accession and voucher numbers).

### Phylogenetic analyses

Multiple sequence alignments of the 28S rRNA and COI genes were constructed using Mafft (vers. 7.419; Katoh et al. 2002; Nakamura et al. 2018) with default parameter settings. The mitochondrial 16S rRNA sequences were aligned using the program Muscle (vers. 3.8.31; Edgar 2004) with default parameter settings. Alignments were visualized using Seaview (vers. 3.2; Gouy et al. 2009). COI sequences were translated into amino acids. The program Gblocks (vers. 0.91b; Castresana 2000; Talavera and Castresana 2007) was applied to the 16S and 28S rRNA gene alignments to check for unambiguously aligned sites. Proposed exclusion sites were reviewed, adjusted, and subsequently removed (alignments before and after editing are deposited at https://doi.org/10.5281/zenodo.3613958). Sequences available from NCBI GenBank for in-group taxa (*C. glabrum* (Montagu, 1803) and *C. glabellum* (A. Adams, 1868)), as well as for out-group taxa were added (Table 3). Outgroups were assigned based on the recent molecular phylogenetic analyses by Golding (2014a, b) and Criscione and Ponder (2013).

**Table 3.** List of included Caecidae and outgroup taxa for phylogenetic analyses downloaded from NCBI GenBank (including accession numbers).

Genus	Species	Author	GenBank number		
			28S rRNA	16S rRNA	COI
* Caecum *	* glabrum *	(Montagu, 1803)		FN820514	
* Caecum *	* glabellum *	(A. Adams, 1868)	AB930352		AB930481
* Elachorbis *	* subtatei *	(Suter, 1907)	KC110005	KC109953	KC439807
* Aenigmula *	* criscionei *	Golding, 2014	KC439956	KC439911	KC439788
* Pseudomerelina *	* mahimensis *	(Melvill, 1893)	KC439943	KC439894	KC439772
* Auricorona *	* queenslandica *	Golding, 2014	KC439953	KC439907	KC439786
* Nozeba *	* topaziaca *	(Hedley, 1908)	KC439952	KC439906	KC439784
* Clenchiella *	* minutissima *	(Wattebled, 1884)	KC439803	KC109947	KC109999
* Calopia *	* imitata *	Ponder, 1999	KC439790	KC439912	KC439957
* Calopia *	* laseroni *	Ponder, 1999	KC439792	KC439914	KC439959

Two combined data sets were generated: (1) a concatenated alignment of all three marker genes and (2) a concatenated alignment comprising only the mitochondrial 16S rRNA gene, and COI. The data were combined into single matrices using P4 (Foster 2004). The combined data sets were then partitioned by gene and COI codon position.

Maximum likelihood (ML) and Bayesian inference (BI) were used to construct the phylogenetic tree from single genes and from combined and partitioned alignments. For each alignment jModelTest2 (vers. 2.1.10; Darriba et al. 2012) was run and the calculated likelihood scores weighted under the Akaike Information criterion (AICc) (Hurvich and Tsai 1989) which suggested GTR+I+G as the best fitting model. ML was performed using IQ-TREE (multicore vers. 1.6.7.1 for Linux 64-bit; Nguyen et al. 2014) with the GTR+G4+FO model (equivalent to GTR+G in RAxML vers. 8.2; Stamatakis 2014) with 300 bootstrap replicates. Bayesian MCMC analyses were performed using the program MrBayes (vers. v.3.2.6; Huelsenbeck and Ronquist 2001) with the same model. The Bayesian analyses were run in duplicates by default, with each run having four parallel Markov chains (MCMC) to estimate posterior probability support. Each chain was run for 5 million generations, sampling trees every 1000^th^ generation. Sampled trees were combined into a consensus tree after the first 1000 sampled trees (1000000 generations), considered as ‘burn-in’, were discarded. A general time-reversible model of nucleotide substitutions with a gamma-distribution of among-site rates (GTR+G) was used for the ML analyses. All trees were visualized and annotated using Figtree (vers. 1.4.4; Rambaut 2007). Boostrap support values (BS) > 85% and posterior probabilities > 0.95 were considered statistically significant.

### Species delimitation and characterization based on molecular data

Four different methods of species delineation were used with both the COI and 16S rRNA gene mitochondrial data sets. The Automatic Barcode Gap Discovery (ABGD) webserver was used to partition the data set into putative species based on the calculated gap between intra- and interspecific genetic differences (https://bioinfo.mnhn.fr/abi/public/abgd/abgdweb.html; Puillandre et al. 2012). J ModelTest2 (vers. 2.1.10; Darriba et al. 2012) was applied to the uncorrected COI and 16S rRNA gene alignments and the parameters were weighted under the corrected Akaike Information criterion (AICc) (Hurvich and Tsai 1989). For both alignments the Jukes-Cantor (JC69) as well as Kimura (K80) model showed to be within the 100% confidence interval however, K80 had slightly higher likelihood scores. Both models were applied with the default settings (TS/TV = 2.0, relative gap width = 1.5, Pmin = 0.001, and Pmax = 0.10) on the uncorrected COI and 16S rRNA gene alignments.

To evaluate haplotype connectivity, we generated haplotype networks based on the COI as well as the 16S rRNA gene sequence alignment using the software TCS (vers. 1.21; Clement et al. 2000) using the standard 95% parsimony setting. Ambiguous sites in both alignments were removed to prevent the creation of artificial haplotypes.

The bPTP web server (https://species.h-its.org/) was used to conduct the Bayesian implementation of the PTP model for species delimitation (Zhang et al. 2013) on the optimal ML trees of the individual and the combined COI and 16S rDNA datasets. We applied the default settings with 100000 generations, thinning for each 100^th^ sample with a burn-in of 10% and checked for convergence of the MCMC chains of each run. Posterior probability (PP) support values above 0.95 were considered as strong support.

For the General Mixed Yule-Coalescent model (GMYC) (Pons et al. 2006), ultrametric trees from the COI, 16S rRNA gene, and combined COI and 16S rRNA gene data were obtained using a time calibrated Bayesian evolutionary analysis in Beast (vers. 1.7.4; Drummond and Rambaut 2007). For the tree prior, we used a Yule process and two fossil records, *Caecum cooperi* and *Caecum imbricatum* [2.58–1.80 myr] (Mansfield 1930; Cooke 1936; Ward and Blackwelder 1987) and the in-group *Caecum* [50–55 myr] (Goedert and Raines 2016) with a lognormal distribution (logL). The analysis was run with the GTR substitution model and under a strict clock assumption. The analysis was started from a random tree and two Markov chains run for 10 000 000 generations with a sampling frequency of 1000. Convergence of the chains was checked in Tracer (vers. 1.7.4.; Rambaut et al. 2018) and effective sampling sizes (ESS) were confirmed as > 200 for all values (Rambaut et al. 2018). The first 10% of sampled trees were removed as burn-in and the trees were combined in TreeAnnotator (vers. 1.7.4.; Drummond et al. 2012) using the maximum clade credibility option and mean node height. The ultrametric trees were uploaded to the web server (https://species.h-its.org/gmyc) for single, as well as, multiple threshold GMYC analyses.

The software QUIDDICH (vers. 1.0.0; Kühn and Haase 2019) was used to identify the diagnostic molecular characters of morphologically cryptic species. We extracted diagnostic characters of type 1 (i.e., characters, which distinguish each individual of the investigated species from other caecids with a fixed character state in the investigated species) and of type 2 (i.e., characters, which distinguish each individual of the investigated species from all other caecids, but vary also within the investigated species) from the COI, 16S rRNA gene and 28S rRNA gene alignments of the same dataset also used for the species delineation and phylogenetic analyses.

## Results

### Molecular phylogeny and primary species hypothesis

**Figure 3.**
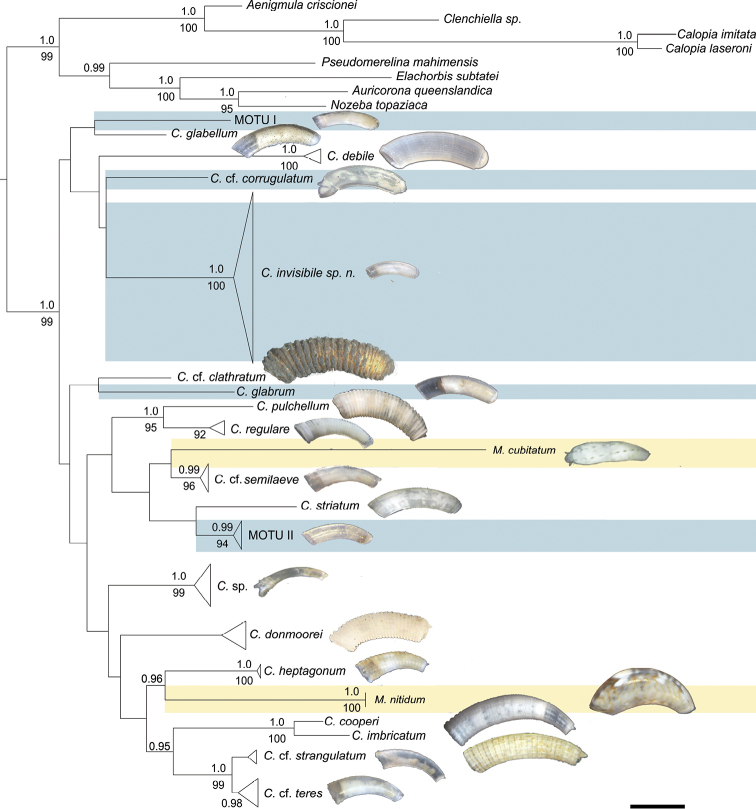
Optimal ML tree of the concatenated 28S rRNA, 16S rRNA and COI genes partitioned by genes and COI codon positions. Bootstrap values (below nodes) of the ML analysis are shown for values > 80% and posterior probability support (above nodes) of the BI analysis are shown for values > 0.95. Specimens previously classified as *Meioceras* are indicated in yellow color. Smooth, translucent specimens, lacking diagnostic features and summarized in the ´*Caecum glabrum*-like complex` are indicated in blue. *C.* = *Caecum*, *M.* = *Meioceras*, MOTU I/ MOTU II = molecular operational taxonomic unit within the ´*Caecum glabrum*-like complex`. Figured specimens are all to the same scale. Scale bar: 1 mm.

In our phylogenetic analyses Caecidae form a well-supported clade (1.0 PP, 99% BS; Fig. 3). The two established genera *Caecum* and *Meioceras*, however, are not recovered as reciprocally monophyletic but instead species of *Meioceras* group among *Caecum* species in different parts of the tree (Fig. 3, taxa highlighted in yellow): *M. nitidum* sister to *C. heptagonum* (0.96 PP), and *M. cubitatum* sister to C. cf. semilaeve (no statistical support). The phylogeny groups the Caecidae into 21 clades which show moderate to high support values ranging from 0.95 PP/85% BS to full support (Fig. 3). Other clades are only statistically supported by one analysis (*C. regulare*, 92% BS) or do not have statistical support (*C. donmoorei*). The sister group relationships of *C. pulchellum* and *C. regulare* (1.0 PP, 95% BS), and *C. cooperi* and *C. imbricatum* (1.0 PP, 100% BS) are well supported; otherwise, deeper nodes and higher-level relationships among clades are not supported. In agreement with the molecular data, *C. pulchellum* and *C. regulare* as well as *C. cooperi* and *C. imbricatum* show morphological similarities in shell ornamentation and microsculpture. Inconspicuous specimens with smooth shells and few characters that were morphologically ascribable to *C. glabrum* or the American Pacific look-alikes like *C. glabriforme* are polyphyletic and form four lineages separated by branches of comparable length to morphologically distinct species (Fig. 3, highlighted in blue). These lineages are distinct from *C. glabrum* from the North Atlantic (Table 3) included in the analyses, indicating the presence of morphologically cryptic species in this ‘*C. glabrum* species complex’.

### Molecular species delineation

**Figure 4.**
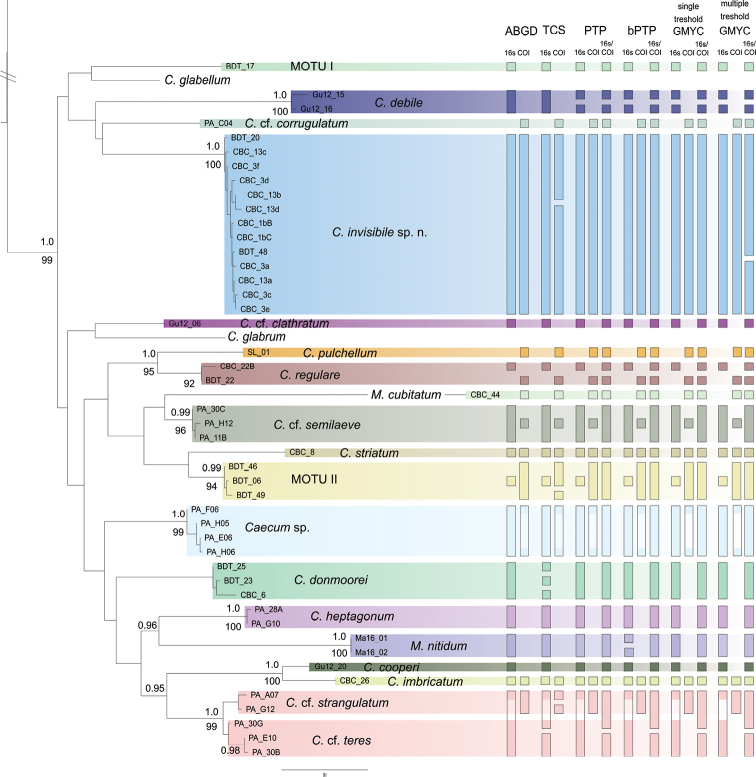
Molecular based species delimitation of Central American Caecidae. Guide tree used for PTP and bPTP based on the optimal likelihood tree of the concatenated three-marker dataset. Color codes indicate our preliminary species hypothesis derived from the phylogenetic tree. Color bars reflect the species delimitation suggested by the four consulted species delimitation programs (including ML and Bayesian implementation for PTP and single and multiple threshold for GMYC). Bars are missing where no sequence data obtained.

The methods that were used for species delineation are largely congruent with regard to the assignment of taxa to molecular operational taxonomic units (MOTUs), however individual analyses deviate and evidently differences occur due to incomplete sampling of one of the markers (Fig. 4, Table 1). Both PTP/ bPTP and GMYC (single threshold) delimit 21 MOTUs for the concatenated dataset of COI and 16S rRNA genes (excluding the species whose sequences were retrieved from NCBI GenBank, i.e. North Atlantic *C. glabrum* and Japanese *C. glabellum*). These results are in concordance with the preliminary species hypotheses based on morphological investigation and the molecular phylogenetic tree (Fig. 3) with the exception of additional splits of *C. debile* and *C. regulare* into two distinct MOTUs each. Caecum cf. teres resulted in a single species for 16S rDNA alone, and the Bayesian implementation of bPTP split *M. nitidum* into two separate species based on the 16S rRNA genes (however, support value for the split is 0.501%). The multiple threshold analyses in GMYC additionally splits *C. invisibile* sp. nov. of the ‘*C. glabrum* complex’ into two MOTUs, as does TCS but into differing entities. In analyses of individual datasets (numbers not directly comparable due to missing data) ABGD identified 15 MOTUs in our 16S rRNA gene dataset (Fig. 4), while the COI dataset resulted in a hypothesis of 10 MOTUs independent of the application of the JC69 or the K80 model. In comparison to the other methods, TCS appears to oversplit MOTUs (see e.g., TCS analyses of the COI of *C. donmoorei* in Fig. 4). The algorithm of this haplotype-network software splits the 16S rDNA dataset into 19 independent haplotype networks, while it recovered 13 networks for the COI dataset (Fig. 4). Additionally, TCS also splits MOTU II of the ‘*C. glabrum*-like complex’ into two networks and *C. donmoorei* into three independent networks based on 16S rRNA sequence data. Haplotype networks divided C. cf teres and C. cf. strangulatum into two unconnected networks. However, the split is not congruent with the two monophyletic sister populations of the species tree (Fig. 4). In summary, we consider only splits relevant, which are supported by at least two different analyses or markers, singular deviating signal might either resemble errors in analyses or might be informative in population analyses (for more details see remarks in Systematics section).

### Taxonomy of Central American Caecidae

#### Systematics

**Class Gastropoda Cuvier, 1797**

**Family Caecidae Gray, 1850**

##### Genus Meioceras

Taxon classificationAnimaliaLittorinimorphaCaecidae

Carpenter, 1859

00633BAB-FD8E-58A0-8D40-2DE5781D2228

###### Type species.

*Caecum nitidum* Stimpson, 1851 from Florida by subsequent designation, Carpenter (1859): 438.

Based on the molecular phylogeny, specimens identified as *Meioceras nitidum* and *M. cubitatum* both group among *Caecum* species and should therefore be transferred to this genus. However, considering that only one *M. nitidium* is statistically supported, in the interest of taxonomic stability this finding is pending further molecular studies, once additional material is available, preferably including material from the type localities.

##### Meioceras nitidum

Taxon classificationAnimaliaLittorinimorphaCaecidae

(Stimpson, 1851)

4BE1E04C-B613-54E5-AF61-27316A2F3EF4

Caecum nitidum Stimpson, 1851 in Stimpson (1851a): 112. Type locality: Florida.
Caecum lermondi Dall, 1924: 7; Caecum rotundum de Folin, 1868: 49, pl. 5, fig. 2; Meioceras bitumidum de Folin, 1869: 9, fig. 4; Meioceras carpenteri de Folin, 1869: 8, 9, fig. 3; Meioceras cingulatum Dall, 1892: 302, pl. 16, figs 6, 7; Meioceras contractum de Folin, 1874: 213, t. 2, pl. 4, fig. 7; Meioceras coxi de Folin, 1869: 13, fig. 9; Meioceras crossei de Folin, 1869: 11, 12, fig. 7; Meioceras deshayesi de Folin, 1869: 11, fig. 6; Meioceras elongatum de Folin, 1881: 17, pl. 1, fig. 9; Meioceras fischeri de Folin, 1870: 188, pl. 26, figs 3, 4; Meioceras imiklis de Folin, 1870: 189, pl. 26, figs 5, 6; Meioceras leoni Bérillon, 1874: 251, pl. 5, fig. 3; Meioceras moreleti de Folin, 1869: 10, fig. 5; Meioceras subinflexum de Folin, 1869: 165, pl. 23, fig. 8; Meioceras undulosum de Folin, 1869: 12, fig. 8.

###### Material examined.

**Figure 5.**
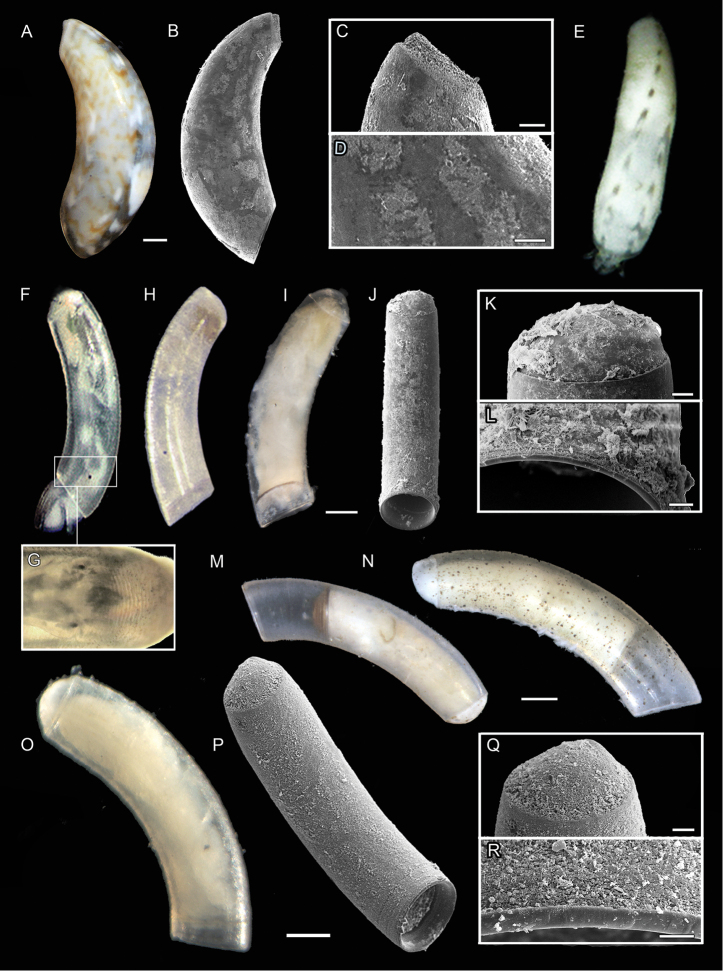
**A–D***Meioceras nitidum*, specimen MNHN-IM-2013-72087 **A** light microscopic picture **B**SEM scan **C**SEM close-up of mucro and **D** microsculpture **E***M. cubitatum*, specimen USNM 1618851 **F, G**C. cf. corrugulatum, specimen USNM 1618861 **F** light microscopic picture **G** light microscopic close-up of microstructure **H** MOTU II, specimen USNM 1618853 **I–L** MOTU I, specimen ZSM-Mol-20200039 **I** light microscopic picture **J**SEM scan **K**SEM close-up of mucro and **L** microsculpture **M***C. glabrum*, specimen ZSM-Mol-20200096 **N***C. glabellum* auctt. non Adams, specimen ZSM-Mol-20200074 **O–R***C. invisibile* sp. nov., holotype ZSM-Mol-20100320 **O** light microscopic picture **P**SEM scan **Q**SEM close-up of mucro and **R** microsculpture. Scale bars: 10 µm (**R**); 20 µm (**C, K, L, Q**); 50 µm (**D, E**) 100 µm (**I, J, O, P**); 200 µm (**A, B, M, N**).

French Antilles • 1 (Fig. 5A–D); Martinique, Anse Noir; 14.528, -61.088; depth 6 m; 6 Sep 2016; MNHN Madibenthos exped.; Stat. AB102; GenBank: MT704298, MT731714; MNHN-IM-2013-72087a • 1; same collection data as for preceding; GenBank: MT704299, MT731715; MNHN-IM-2013-72087b.

###### Shell morphology.

Shell translucent, glossy. Light brown zig-zag pattern covering entire shell in rings with irregular white dorsal patches (Fig. 5A). Bulbous tube, tapering towards aperture and posterior end. Maximum width at about one third of shell length. Slightly more bowed towards aperture. Septum flat, with triangular, pointed mucro (Fig. 5C). No sculpture or microsculpture diagnostic features (Fig. 5D).

###### Remarks.

*Meioceras* and in particular “*M. nitidum*” has a complex taxonomic history involving at present 16 synonyms and several reallocations between *Meioceras* and *Caecum* (MolluscaBase 2019). Vannozzi (2017) highlighted the problems with the ambiguous type specimen of *M. nitidum* (Stimpson 1851a), which encouraged multiple novel descriptions (e.g., Carpenter 1858–1859; De Folin and Périer 1867), nowadays recognized as synonyms. Our two investigated specimens from Martinique are consistent with the meagre original description by Stimpson (1851a) based on specimens from Florida and several redescriptions based on material from the Caribbean and southern America, now all accepted as *M. nitidum* (*M. nitidum* see Bandel 1996: 99, pl. 5, figs 1–7; *M. contractum* see de Folin and Périer 1875: pl. 9, fig. 7). Our specimens differ morphologically from several “*M. nitidum*” specimens from Central American waters all of which were described as a different species in the past but later synonymized with *M. nitidum* acknowledging intraspecific variability (i.e., *M. elongatum*, holotype accessible through the online catalogue of the MNHN (MNHN-IM-2000-32923) and *M. subinflexum* (see de Folin and Périer 1867: pl. 23, fig. 8). Molecular comparison of specimens spanning the morphological and geographical range is needed to clarify the species status and distribution of the species.

##### Meioceras cubitatum

Taxon classificationAnimaliaLittorinimorphaCaecidae

de Folin, 1868

AFFB720D-5201-5F7A-AA55-F649750C3A37

Meioceras cubitatum de Folin, 1868 in De Folin and Périer (1867–1871): 50, pl. 5, fig. 4. Type locality: Baie de Bahia [Bahia Bay, Brazil].
Caecum cubitatum (de Folin, 1868): 19; Meioceras tenerum de Folin, 1869: 24.

###### Material examined.

Belize • 1 (Fig. 5E); Carrie Bow Cay; 16.8021, -88.0767; depth 31 m; 22 Jan 2010; USNM Belize 2010 exped.; Stat. CBC15; DNA voucher; GenBank: MT727067, MT731709; USNM 1618851.

###### Shell morphology.

Shell opaque white and solid. Mottled grayish pattern over whole shell, two rows of distinct brown dashes along dorsal side (Fig. 5E). Specimen approx. 2 mm. Tube not evenly curved but appears bulbous and is rounded strongly towards aperture, decreasing towards mucro. Mucro thin and sharp.

###### Remarks.

Our molecular phylogenetic results delimited *M. cubitatum* as a separate species, despite similarities to *M. nitidum* in its bulbous shell shape and pattern. Surprisingly, our molecular analyses do not retrieve these morphologically similar *Meioceras* species as a monophyletic entity but suggest independent origin within *Caecum*. Morphological differences towards *M. nitidum* (characterized above) are a more slender shell with more pronounced curvature towards the anterior end and the opaque color of the present individual. We assigned the specimen to *Meioceras cubitatum* sensu de Folin, 1869 from Bahia, Brazil (dos Santos Gomes and Absalão 1996: 523, figs 12–15; De Folin and Périer 1867: pl. 5, fig. 4; Redfern 2001: fig. 178A, B) = *Meioceras cornucopiae* Carpenter, 1859 (from the West Indies, exact type locality unknown) sensu Lima et al. (2015: 3, fig. 7). Nevertheless, this *Meioceras* species likely should also be reallocated to the genus *Caecum* based on the results of our phylogenetic analyses.

##### Genus Caecum

Taxon classificationAnimaliaLittorinimorphaCaecidae

J. Fleming, 1813

D0B6A5C4-AA98-576F-8BB2-7B7223396984

###### Type species.

*Dentalium trachea* Montagu, 1803 from England by subsequent designation, Gray 1847: 203.

### Cryptic lineages revealed in molecular analyses

Twenty-four specimens from Central American waters are smooth and glossy without ornamentation except for occasional growth lines (i.e., possess few shell characteristics), but vary in adult shell length between 0.7 and 2.5 mm (Figs 3, 5F–L, O–R). Morphologically, these specimens all closely resemble *Caecum glabrum* (Montagu, 1803) which is one of the best-known species of caecids, and abundant in the northern Atlantic (Montagu 1803; Wood and Harmer 1848; Götze 1938; Chambers 2009). *Caecum glabrum* was originally described from Biddlesford Bay and Barnstable, Devon, England, the included sequences from GenBank (see Table 3) originates from specimens collected in Norway, but own unpublished data from Roscoff, northern France, supports the wide distribution range of *C. glabrum* along European coastlines based on molecular data. We refer to cryptic species with simple shells lacking characteristic features as ‘*Caecum glabrum*-like’ species complex. In previous works, specimens similar to *C. glabrum* have also been described from the Pacific (*C. glabellum* as *Brochina glabella* A. Adams, 1868 from Akashi, Japan, *C. glabriforme* Carpenter, 1857 and *C. corrugulatum* Carpenter, 1857, both from Mazatlán, Mexico and *C. parvulum* de Folin, 1867 from Panama Bay, Brazil), have been reported and described from Japan, Hawaii, and central America (Carpenter 1855–1857; Adams 1868; Lightfoot 1993b; Pizzini et al. 2007, Takano and Kano 2014). Our study clearly shows an independent evolutionary origin of *C. glabrum* from the northeast Atlantic and *C. glabellum* from Japan, and the cryptic *C. glabrum*-like MOTUs from central America (see Fig. 3, ‘*C. glabrum*-like-complex’ highlighted in blue color). Our molecular species delineation revealed a minimum of four cryptic MOTUs (see above, Fig. 4).

#### Caecum cf. corrugulatum

Taxon classificationAnimaliaLittorinimorphaCaecidae

Carpenter, 1857

2CAE5AA9-3E97-5FA4-889D-EDE150342781

Caecum corrugulatum Carpenter, 1857: 327, pl. 37, figs 375, 1547. Type locality: Mazatlán, 1 sp. off Chama [Mexico].

##### Material examined.

Panama • 1 (Fig. 5F, G); Achotines; 7.6207, -80.0013; depth 12 m; 29 Feb 2016; USNM Achotines2016 exped.; Stat. PA14; DNA voucher; GenBank: MT727069, MT731722; USNM 1618861.

##### Shell morphology.

Shell color whitish translucent. Tube regularly curved, shape equal in width but bears prominent edge at transition to septum (Fig. 5F). Septum round and blistered lacking a mucro. Aperture equally wide as tube with straight edge. Sculpture appears completely smooth but shows fine concentric ribs at higher magnification (Fig. 5G).

##### Remarks.

We assigned the specimen collected in the Pacific coast of Panama to *C. corrugulatum* based on the description of Carpenter (1858–1859) who already highlighted its similarity with another inconspicuous species (*C. glabriforme*). Both species are described from the same geographic area (Mazatlán, Pacific coast of Mexico) and resemble the *C. glabrum*-like type: translucent, blistered septum without mucro, smooth, however slightly bigger than the eponymous *C. glabrum* from European waters. *Caecum corrugulatum*, can be distinguished by microsculptural concentric wrinkles, which could be observed with higher magnification in our specimen. So far, only *C. glabriforme* was recorded in Pacific Panama (Lightfoot 1993b) and recollection at the type locality is needed to 1) confirm the validity of both co-occurring species and reject conspecificity and 2) to confirm their putative distribution range from Mexico to Panama and exclude the possibility of further cryptic species among *C. glabriforme* and *C. corrugulatum* species along the Pacific Coast of Central America (as discovered herein for the Atlantic Coast, see below).

#### Caecum invisibile

Taxon classificationAnimaliaLittorinimorphaCaecidae

Egger & Jörger sp. nov.

9A04C84A-44F1-5193-8EC0-7E6F8C0E0A99

http://zoobank.org/4183679F-44F4-4817-A2E1-7325687E5F0A

##### Material examined.

***Holotype*** Belize • 1 (Fig. 5O–R); Carrie Bow Cay; 16.8015, -88.0790; depth 10 m; 14 Jan 2010; USNM Belize2010 exped.; Stat. CBC1b; DNA voucher; DNA bank: r462p15f2t91; GenBank: MT727055, MT704268, MT731697; ZSM-Mol-20100320. ***Paratypes*** Belize • 1; same data as for holotype; DNA voucher; DNA bank: r462p14f2t91; GenBank: MT727054, MT704267, MT731696; ZSM-Mol-20200109. Belize • 2; same data as for holotype; ZSM-Mol-20200111, ZSM-Mol-20200112. **Other material** Belize • 10; same data as for holotype; DNA voucher; GenBank: MT727056–MT727065, MT704269–MT704279, MT731698–MT731707; USNM 1618839, USNM 1618840, USNM 1618841, USNM 1618842, USNM 1618843, USNM 1618846, USNM 1618847, USNM 1618848, USNM 1618849. Panama • 1; Bocas del Toro; 9.2140, -81.9318; depth 8.5 m; 5 Jun 2010; USNM BRS2010 exped.; Stat. BRS104; DNA voucher; GenBank: MT727049, MT704264, MT731689; USNM 1618856. • 1; Bocas del Toro; 9.3507, -82.1724; depth 3 m; 13 Jun 2010; USNM BRS2010 exped.; Stat. BRS200; DNA voucher; GenBank: MT727052, MT731694; USNM 1618859.

##### Molecular diagnostic characters.

see Table 4.

**Table 4.** Type 1 characters and type 2 characters (Kühn and Haase 2019) for COI, 16S rRNA and 28S rRNA sequence data (no type 2 characters for 16S rRNA and 28S rRNA present) of *C. invisibile* sp. nov.

COI				16S rRNA				28S rRNA			
Type 1		Type 2		Type 1		Type 2		Type 1		Type 2	
Position	States	Position	States	Position	States	Position	States	Position	States	Position	States
15	A	501	T	7	C			378	G	595	CA
171	G	450	G	9	C			392	A		
267	G			32	T			414	T		
279	T			93	G			426	C		
300	G			97	T			515	T		
				104	T			598	A		
				191	T			612	G		
				223	G			649	A		
				227	G			664	T		
				247	C						

##### Morphological description.

All investigated specimens were very similar in appearance, with little or no variation in shell morphology. Shell completely translucent. Length 0.8 mm long, width 0.2 mm (holotype, Fig. 5O). Tube regularly curved, shape equal in width but bears prominent edge at transition to septum, edge with smaller diameter. Septum round and blistered lacking a distinct mucro. Septum slightly inclining towards the left, dorsal side in holotype with slight variation between the specimens. Aperture equally wide as tube with straight edge. Sculpture appears smooth, only with faint growth lines (Fig. 5R). Whitish translucent body visible through translucent shell. Operculum translucent, slightly tinted yellowish. Radula formula shows taenioglossate pattern 2.1.1.1.2. with very small central rhachidian tooth. Large lateral teeth oriented towards the rhachidian tooth. Marginal teeth finer, outer marginal teeth are scoop-like curved. All the specimens investigated are adults based on the cylindrical shape of the tube and the shape of the aperture showing a reflected lip without cutting edge, which is normally present in immature specimens.

##### Etymology.

The Latin adjective *invisibile* (invisible, unable to be seen) refers to the minute size of specimens, the translucent color of its shell, its hidden lifestyle between sand grains, and its taxonomic crypsis.

##### Distribution.

Type locality: Carrie Bow Cay, Belize. (16.8015°N, -88.0790°W, -10 m). Distributed in Central American Atlantic from Carrie Bow Cay, Belize to Bocas del Toro, Panama. Interstitial in coarse biogenic sediments (calcareous sand and shell hash), shallow subtidal at ten meters’ depth.

##### Remarks.

*Caecum invisibile* sp. nov. is described as a new species based on molecular diagnostic characters, which show it as distinct from the European *C. glabrum* (Fig. 5M), as well as the morphologically similar *C. corrugulatum* (Fig. 5F, G) from the Central American Pacific and *C. glabellum* from Japan (Fig. 5N).

###### MOTU I

**Material examined.** Panama • 1 (Fig. 5I–L); Bocas del Toro; 2010; USNM BRS2010 exped.; DNA voucher; DNA bank: r462p13f2t91; GenBank: MT704263, MT731688; ZSM-Mol-20200039.

**Morphological characterization.** Shell size 1.3 mm long, 0.3 mm wide. Translucent, with whitish body. Tube regularly curved and equal width. Septum hemispherical (Fig. 5K). Aperture straight, with lip indicating an adult specimen. Operculum brownish. No sculpture or microsculpture diagnostic features (compare Fig. 5L).

**Remarks.** MOTU I is highly similar to the European *C. glabrum* (Fig. 5M) and *Caecum invisibile* sp. nov. (Fig. 5O–R). However, MOTU I shows some small morphological differences such as a bigger shell size and a tiny rim at the aperture (Fig. 5L) which is absent in *C. glabrum*. The septum is further completely round and blistered (Fig. 5K), whereas the one of *Caecum invisibile* sp. nov. slightly inclines (Fig. 5Q). MOTU I is based on a singleton and an incomplete molecular dataset, lacking COI sequence data. Additional material from the same locality is necessary to justify proper species description in future research.

###### MOTU II

**Material examined.** Panama • 1 (Fig. 5H); Bocas del Toro; 9.4333, -82.347; depth 3 m; 5 Jun 2010; USNM BRS2010 exped.; Stat. BRS101; DNA voucher; GenBank: MT727046, MT704262, MT731687; USNM 1618853. • 1; Bocas del Toro; 9.3507, -82.1724; depth 15 m; 13 Jun 2010; USNM BRS2010 exped.; Stat. BRS110; DNA voucher; GenBank: MT727051, MT731693; USNM 1618852. • 1; Bocas del Toro; 9.3507, -82.1724; depth 3 m; 13 Jun 2010; USNM BRS2010 exped.; Stat. BRS200; DNA voucher; GenBank: MT727053, MT731695; USNM 1618860.

**Morphological characterization.** Shell size unknown. Translucent, with translucent body. Tube regularly curved, slightly increasing in diameter towards aperture. Septum round, slightly flattened (Fig. 5H). Aperture straight. No sculpture visible, microsculptural data missing.

**Remarks.** MOTU II is based on the molecular data of three specimens; however, we unfortunately lack SEM scans and thus microsculptural data of the shell and light microscopic images are only available for one specimen (Fig. 5H). This specimen is a juvenile, and due to uncertainty with regards to adult ornamentation of the shell, and its possible identity with an already described species, we refrain from providing a formal description based on the available material only.

### Adding barcodes to known Central American Caecidae

#### Caecum heptagonum

Taxon classificationAnimaliaLittorinimorphaCaecidae

Carpenter, 1857

ADB50B90-7DE4-511D-BC12-F4A67DC2B51D

Caecum heptagonum Carpenter, 1857: 319, t. 1524. Type locality: Mazatlán [Mexico].

##### Material examined.

**Figure 6.**
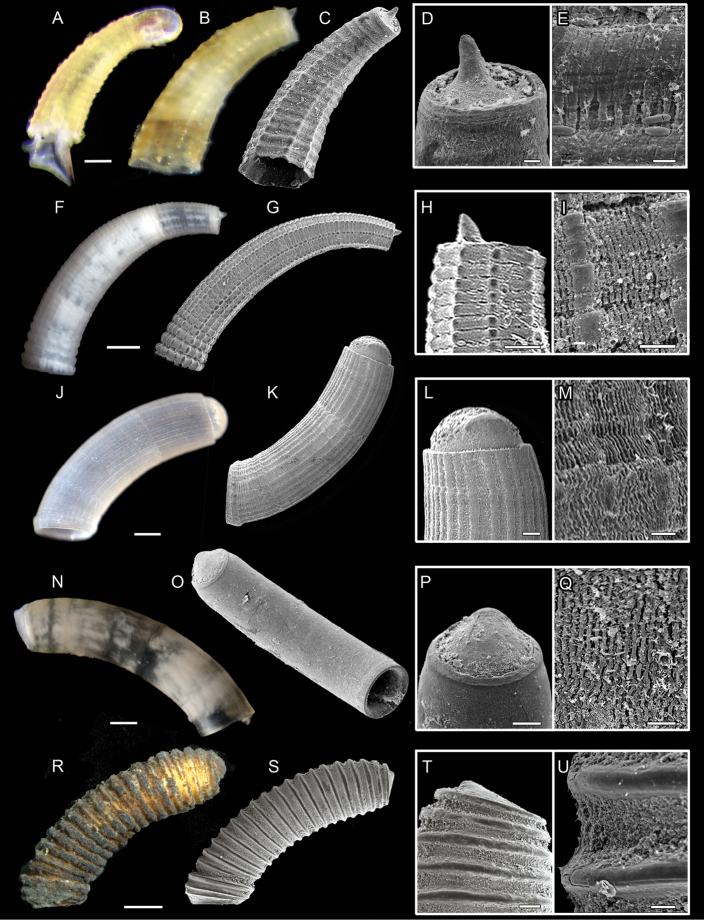
**A–E***Caecum heptagonum***A** specimen USNM 1618866 juvenile specimen with larval shell still attached **B–E** specimen ZSM-Mol-20200030, juvenile specimen already resembling closely the adult form **B** light microscopic picture **C**SEM scan **D** close-up of mucro and **E** microsculpture **F–I***C. cooperi*, specimen MNHN-IM-2019-32 **F** light microscopic picture **G**SEM scan **H** close-up of mucro and **I** microsculpture whole specimen and close-up of mucro and microsculpture **J–M***C. debile*, specimen MNHN-IM-2019-27 **J** light microscopic picture **K**SEM scan **L** close-up of mucro and **M** microsculpture **N–Q***C. striatum*, specimen ZSM-Mol-20100322 **N** light microscopic picture **O**SEM scan **P** close-up of mucro and **Q** microsculpture **R–U***C. clathratum*, specimen MNHN-IM-2019-17 **R** light microscopic picture **S**SEM scan **T** close-up of mucro and **U** microsculpture. Scale bars: 10 µm (**D, E, I, Q**); 20 µm (**H, M, P, U**); 100 µm (**A, B, C, L, N, O, T**); 200 µm (**F, G, J, K** whole specimen); 300 µm (**R, S**).

Panama • 1 juv. (Fig. 6B–E); Achotines; 7.6349, -79.9968; depth 10 m; 6 Mar 2016; USNM Achotines2016 exped.; Stat. PA23a; DNA voucher; DNA bank: r462p4f2t91, GenBank: MT704283, MT731717; ZSM-Mol-20200030. • 1 juv. (Fig. 6A); same collection data as for preceding; DNA voucher; GenBank: MT704291, MT731726; USNM 1618866. • 1 juv.; same collection data as for preceding; ZSM-Mol-20200116. • 1 juv.; same collection data as for preceding; ZSM-Mol-20200117. • 1 juv.; Achotines; 7.6346, -79.9965; depth 10 m; 6 Mar 2016; USNM Achotines2016 exped.; Stat. PA23b; ZSM-Mol-20200115.

##### Shell morphology.

In juvenile specimens, shell fragile, translucent brownish color. Tube doubles diameter towards aperture, with a moderate curvature in anterior half, increasing distally in curvature. Septum level beneath cutting plane, slightly rising towards mucro (Fig. 6D). Mucro slender finger-like shape (Fig. 6D). Aperture fragile and partly broken. Shell sculptured by seven longitudinal ridges with transverse ribs crossing, knobs at intersections, ridges less prominent towards posterior. Microsculpture of fine rugose longitudinal stripes, noticeably increasing in width on transversal rings in comparison to interspaces (Fig. 6E).

##### Remarks.

Due to the characteristics of the heptagonal tube with the transversal rings, considered unique among caecids (Lightfoot 1993a), the investigated specimen could be unambiguously assigned to *C. heptagonum*. However, illustrations of *C. heptagonum* indicate a very thick shell with distinct differentiated aperture with inner bulge forming a round opening instead of the outer polygonal shape (Keen 1974; Pizzini et al. 1998: 142, figs 1–13) including an inner bulge in the aperture, forming a round opening instead of the outer polygonal shape which is absent in the rather thin and fragile investigated specimens. As our samples only comprised juvenile specimen, however, we can attribute this variation to the unfinished shell state.

#### Caecum imbricatum

Taxon classificationAnimaliaLittorinimorphaCaecidae

Carpenter, 1858

6CB52060-89CB-5414-98DF-2681A2610F89

Caecum imbricatum Carpenter, 1858: 422, pl. 69, fig. 10. Type locality: “W. Indies [Carribean].
Caecum coronatum de Folin, 1867: 50–52, pl. 2, fig. 5; Caecum formulosum de Folin, 1869: 24–125, pl. 11, figs 9, 10 (with three varieties *paucicostata*, *simplex* and *sulcate*); Caecum insigne de Folin, 1867: 52, 53, pl. 2, fig. 4; Caecum sculptum de Folin, 1881: 15, pl. I, figs 1, 2.

##### Material examined.

Belize • 1 (Fig. 1, specimen to the left); Carrie Bow Cay; 16.8037, -88.0769; depth 31 m; 15 Jan 2010; USNM Belize2010 exped.; DNA voucher; GenBank: MT727051, MT704281; USNM 1618850. Panama • 1; Bocas del Toro; 9.4333, -82.3467; depth 3 m; 5 Jun 2010; USNM BRS2010 exped.; DNA voucher; GenBank: MT704261; USNM 1618852. • 1; Bocas del Toro; 9.3222, -82.2215; depth 4.5 m; 5 Jun 2010; USNM BRS2010 exped.; DNA voucher; GenBank: MT727047; USNM 1618854. • 1; same collection data as for preceding; GenBank: MT727048; USNM 1618855.

##### Shell morphology.

Shell opaque, yellowish. Lighter color in interspaces, darker colored ridges of prominent rhombic pattern (Fig. 1, first specimen from left). Tube evenly narrows towards posterior end, which is approximately half as wide in diameter as aperture. Septum flat, triangular shaped, strongly pointed mucro (Fig. 1, first specimen from left). Rhombic pattern consisting of distinct longitudinal ridges crossed by axial ridges, pattern more distinct towards aperture, last row forms bumps at intersections ((Fig. 1, specimen to the left).

##### Remarks.

See remarks on *C. cooperi*. “ after paragraph on shell morphology of *C. imbricatum*.

#### Caecum cooperi

Taxon classificationAnimaliaLittorinimorphaCaecidae

S. Smith, 1860

D9CA2160-302B-5D14-85AC-4ADAC3A1015A

Caecum cooperi S. Smith, 1860: 154–155. Type locality: northern part of Gardiner’s Bay, four or five fathoms [United States].
Caecum costatum A. E. Verrill, 1872: 283, pl. 6, fig. 6, Caecum smithi Cooper, 1872.

##### Material examined.

French Antilles • 1 (Fig. 6F–I); Guadeloupe; 16.3558, -61.5965; depth 2 m; 24 May 2012; MNHN KARUBENTHOS exped.; Stat. GS32; GenBank: MT704297, MT731713; MNHN-IM-2019-32.

##### Shell morphology.

Shell opaque, with whitish diffuse patterns. Size > 2 mm, tube narrow and elongated, curvature increasing towards posterior end (Fig. 6F, G). Septum flat, triangular shaped, strongly pointed mucro (Fig. 6H) similar to mucro in *C. imbricatum*. Prominent and conspicuous shell ornamentation, minimum of 20 longitudinal strings of beads, more pronounced towards aperture (Fig. 6I).

##### Remarks.

Our molecular species delimitation separates *C. cooperi* and *C. imbricatum* into two independent evolving sister species (see Figs 3, 4). This is supported by (minor) morphological differences such as a finer but more pronounced bead-like ornamentation in *C. cooperi* in comparison to flattened squarish and less frequent longitudinal pattern. The included barcodes and distinguishing diagnostic features should help to overcome previous taxonomic uncertainty suggestive of synonymy (see *C. imbricatum* sensu Moore (1972: 888, fig. 6)). The putative synonymy of *C. insularum* Moore, 1969 and *C. imbricatum* (compare *C. imbricatum* sensu Tunnell et al. (2010: 144) = *C. insularum* sensu Moore (1970: 370, fig. 1A, B) needs to be tested by molecular data, ideally based on material recollected from the type localities.

#### Caecum debile

Taxon classificationAnimaliaLittorinimorphaCaecidae

Verrill & Bush, 1900

4879A9EF-1349-5BBA-AD5B-3F392EB24411

Caecum debile Verrill & Bush, 1900: 538. Type locality: Bermuda, Ship Channel and Bailey Bay, in 12 to 40 feet.

##### Material examined.

French Antilles • 1 (Fig. 6J–M); Guadeloupe; 16.3558, -61.5965; depth 2 m; 24 May 2012; MNHN KARUBENTHOS exped.; Stat. GS32 GenBank: MT704295, MT731711; MNHN-IM-2019-27a. • 1; same collection data as for preceding; GenBank: MT704296, MT731712; MNHN-IM-2019-27b.

##### Shell morphology.

Color whitish, slightly translucent. Specimens about 2.0 mm long, 0.5 mm wide. Tube of adult specimen 2.1 mm long, 0.5 mm wide. Tube evenly curved and evenly wide over entire length (Fig. 6J, K). Septum separated by sharp rim from tube and hemispherical without protruding mucro (Fig. 6L). Aperture with protruding ring. Shell structured by longitudinal striae clearly observable via LM. Microsculpture of fine wavy striation (Fig. 6M).

##### Remarks.

The present specimens were assigned to *C. debile* based on the characteristic microsculpture (see Absalão and Gomes 2001). Morphologically, *C. debile* might present a synonym of *C. infimum*, de Folin, 1867 (de Folin and Périer 1867: 26, pl .3, fig. 2) but, in awareness of cryptic species we refrain from synonymizing until *C. infimum* from the type locality is available for molecular analyses. Some species delineation analyses, separate *C. debile* into two independent species (Fig. 4), which might indicate a putative speciation, but more data on the genetic variability is needed to exclude the presence of an artefact in analyses.

#### Caecum striatum

Taxon classificationAnimaliaLittorinimorphaCaecidae

de Folin, 1868

2C0C8B47-B37A-5363-BF08-EF5564A85D83

Caecum striatum de Folin, 1868 in De Folin and Périer (1867–1871): 49, pl. 5, fig. 3 (with variety *obsoleta* de Folin, 1874). Type locality: Baie de Bahia [Bahia Bay, Brazil].

##### Material examined.

Belize • 1; Carrie Bow Cay; 16.8024, -88.0776; depth 19 m; 25 Jan 2010; USNM Belize2010 exped.; Stat. CBC24; DNA voucher; GenBank: MT727061, MT704275; USNM 1618845. • 1 (Fig. 6N–Q); same collection data as for preceding; ZSM-Mol-20100322.

##### Shell morphology.

Shell translucent with mottled ochre and white marbling (Fig. 6N). Size 1.5 mm in length and 0.3 mm in width with thick shell (= 10 µm at aperture). Tube curved regularly with equal width at posterior and anterior end. Blistered, dome-shaped septum with prominent ring separating tube from septum (Fig. 6P). Mucro central, flat and rounded, pointing slightly dorsal, hardly separated from septum. Aperture simple, straight. Sculpture not visible under the light microscope, i.e., shell appears rather smooth despite two faint transversal rings slightly noticeable close to aperture. SEM examination reveals longitudinal and slightly wavy structure (Fig. 6Q).

##### Remarks.

**Figure 7.**
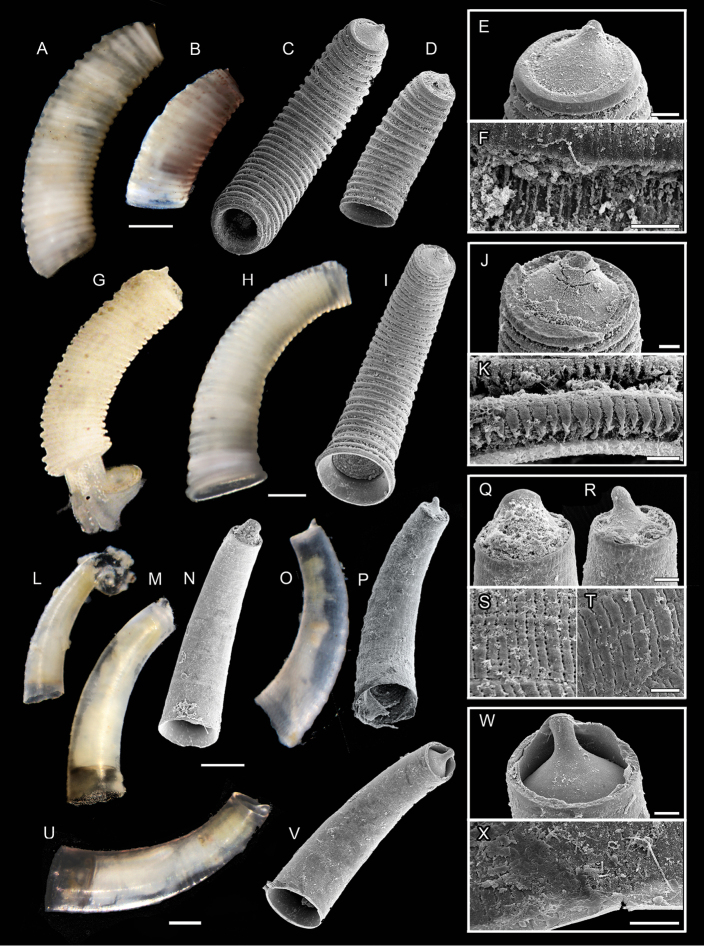
**A–F***Caecum pulchellum***A, C, E, F** specimen ZSM-Mol-20090485 **B, D** juvenile specimen ZSM-Mol-20200118 **A, B** light microscopic pictures **C, D**SEM scans **E** close-up of mucro and **F** microsculpture **H–K***C. donmoorei*, specimen USNM 1618844 **H–K***C. regulare*, specimen ZSM-Mol-20100321, juvenile **H** light microscopic picture **I**SEM scans **J** close-up of mucro and **K** microsculpture **L–T**C. cf. teres and C. cf. strangulatum**L** specimen ZSM-Mol-20200033, juvenile specimen with larval shell still attached **M, N, Q, S** specimen ZSM-Mol-20200032, juvenile **M** light microscopic picture **N**SEM scan **Q** close-up of mucro and **S** microsculpture **O, P, R, T**C. cf. strangulatum, specimen ZSM-Mol-20200038, juvenile **O** light microscopic picture **P**SEM scan **R** close-up of mucro and **T** microsculpture **U–X**C. cf. semilaeve, specimen ZSM-Mol-20200034 **U** Light microscopic picture **V**SEM scan **W** close-up of mucro and **X** microsculpture. Scale bars: 10 µm (**F, S, T, W, X**); 20 µm (**J, K, Q, R**); 50 µm (**E**); 100 µm (**H, I, L–P, U, V**); 200 µm (**A–D**).

*Caecum striatum* was identified based on a comparison with the material collected in the sampling region and dedicated as lectotypes by Absalão and Gomes (2001). Their microsculptural description of the type material does correspond to the longitudinal striation of our investigated specimen. Furthermore, shape, mucro, and the noticeably sharp aperture are identical to our specimen. A comparison with a specimen of *C. striatum* pictured by Pastorino and Chiesa (2014: figs 10–16), also shows the same fine-lined microsculpture as our specimen. Type specimens of three highly similar species, namely *C. johnsoni* Winkley, 1809, *C. antillarum* Carpenter, 1858 and *C. strigosum* de Folin, 1867, are described from Central American waters. Differences can be compared in the reinvestigation of Absalão and Gomes (2001: 20, figs 39–41, 12, figs 11, 12 and figs 7, 8 respectively).

#### Caecum cf. clathratum

Taxon classificationAnimaliaLittorinimorphaCaecidae

Carpenter, 1857

AAA2AAA1-A001-5C88-8BD0-4B422907725D

Caecum clathratum Carpenter, 1857 in Carpenter (1855–1857): 322, pl. 34, figs 269, 1528. Type locality: Mazatlán [Mexico].

##### Material examined.

French Antilles • 1 (Fig. 6R–U); Guadeloupe; 16.2235, -61.5305; depth 1 m; 2 May 2012; MNHN KARUBENTHOS exped.; Stat. GM01; GenBank: MT704294, MT731710; MNHN-IM-2019-17.

##### Shell morphology.

Large, thick shell (3.0 mm length and 0.8 mm width) with and even curvature (Fig. 6R). Color opaque yellow brownish, entire specimen covered in dense dark periostracum. Septum triangular merged with pointed mucro (Fig. 6T). Aperture oblique and constricted. Shell with 21 strong and protruding sharp ribs and deep interspaces narrowing at aperture (Fig. 6R, S). Ribs and interspaces smooth without microsculptural diagnostic features (Fig. 6U).

##### Remarks.

The specimen corresponds to *C. clathratum*, which differs from other ribbed *Caecum* species by its exceptional size, golden color and lack of microsculpture (compare with Lightfoot 1993a: 15, fig. 1 and a syntype collected by Carpenter available through the online catalogue of the Natural History Museum London catalogue number 1857.6.4.1528). However, the specimen is described and known only from the Eastern Pacific. Our herein investigated specimen from the Atlantic might thus present a (morphologically cryptic) sister species new to science, which potentially originated when populations were separated via the formation of the Isthmus of Panama. But molecular data of specimens collected from the Eastern Pacific is required to confirm the molecular identity or justify the description of a new species.

#### Caecum pulchellum

Taxon classificationAnimaliaLittorinimorphaCaecidae

Stimpson, 1851

0F2CAFCE-5D0E-5700-9C81-E18895A72F6B

Caecum pulchellum Stimpson, 1851 in Stimpson (1851b): 36, pl. 2, fig. 3. Type locality: New England, Buzzard’s Bay [New Bedford Harbor, Massachusetts].
Caecum capitanum de Folin, 1874: 227, 228, pl. 9, fig. 8; Caecum conjunctum de Folin, 1867: 46, pl. 4, figs 5, 6, Caecum curtatum de Folin, 1867: 20, pl. 2, figs 4, 5

##### Material examined.

Saint Lucia • 1 (Fig. 7A, C, E, F); Soufriere Bay; 13.8494, -61.0675; depth 8–9 m; 19 Feb 2009; ZSM stuff leg.; DNA voucher; DNA bank: r462p19f2t91; GenBank: MT727074, MT704300, MT731729; ZSM-Mol-20090485. • 1, juv. (Fig. 7B, D); same collection data as for preceding; DNA voucher; DNA bank: r462p20f2t91; ZSM-Mol-20200118.

##### Shell description.

Color opaque whitish, slightly translucent, shell thick (Fig. 7A). Adult specimen 2.1 mm long, 0.5 mm wide. Tube slightly tapering, constricted at aperture, with thickened lip (Figs 7C). Septum slightly lower than posterior end of tube, rising, small and peaked mucro (Fig. 7E). Shell bears 27 squares transverse ribs of even width and equal interspaces, except two or three ribs which meld close to the aperture. Topmost ring sloped towards septum and smaller than others. Fine inconspicuous longitudinal microstriae cover ribs (Fig. 7F). Interspaces covered with organic material, therefore no microsculptural pattern visible.

##### Remarks.

Our investigated specimens agree well with recent descriptions and geographical records of *C. pulchellum* (e.g., Bandel (1996): 112, 113, pl. 6, figs 1–4, 6, pl. 7, figs 1, 2 from Columbia and Curaçao; Lightfoot (1992a): 143, fig. 2). The type specimen, however, is originally described from Buzzard’s Bay in Massachusetts, USA. A molecular comparison of specimen from the Caribbean and northeast America is needed to confirm the distribution range based on morphology.

#### Caecum regulare

Taxon classificationAnimaliaLittorinimorphaCaecidae

Carpenter, 1858

D1E79BAC-82FE-5737-A5B4-7334870E914B

Caecum regulare Carpenter, 1858 in Carpenter (1858–1859): 428–429, pl. 69. Type locality: W. Indies (Woodward) [Caribbean].

##### Material examined.

Panama • 1, juv.; Bocas del Toro; 9.2141, -81.9318; depth 8.5 m; 5 Jun 2010; USNM BRS2010 exped.; Stat. BRS108; DNA voucher; GenBank: MT727050, MT731690; USNM 1618883. Belize • 1, juv. (Fig. 7H–K); Carrie Bow Cay; 16.7911, -88.0761; depth 15 m; 24 Jan 2010; USNM Belize2010 exped.; Stat. CBC22; DNA voucher; DNA bank: r462p17f2t91; GenBank: MT704280, MT731708; ZSM-Mol-20100321. • 1, juv.; same collection data as for preceding; ZSM-Mol-20200114.

##### Shell morphology.

Shell translucent, color white to yellowish (Fig. 7H). Size 1.0 mm long, 0.4 mm wide. Tube curved regularly in moderate angle, tapering towards posterior, widening at aperture, thick lip (10 µm) (Fig. 7H, I). Septum slightly domed. Mucro slender and sharply pointed (Fig. 7J, but tip broken in specimen ZSM-Mol-20100321). Sculpture consists of 32 marked and rounded transverse ribs, narrowing towards septum, with narrow deep interspaces (Fig. 7I). Microsculpture shows longitudinal fusiform lobes covering the ribs (Fig. 7K).

##### Remarks.

We identified the specimens as *C. regulare* by referring to the drawings of Carpenter’s original description (Carpenter 1857; 1858–1859), the syntype material from the Natural History Museum, London, UK accessed through the online catalogue (catalogue numbers 1858.12.9.19, 1858.12.9.20, 1858.12.9.21) and the re-description of Moore (1972): 888, fig. 7. The conspicuous widening of the shell very close to the aperture in our specimen investigated can be interpreted as a character of a sub-adult growth stage (Bandel 1996), thus not contradicting the assignment to *C. regulare*. A literature survey suggests at least one putative synonym of *C. regulare*, resp. *Caecum planum*, de Folin, 1874 (de Folin and Périer 1875: 277, t. 2, pl.10, figs 8, 9). However, the geographical distribution differs (*C. regulare* was originally described from the Caribbean and *C. planum* from Brazil), and there are no evident diagnostic differences to *C. regulare.* Hence, molecular data is needed for clarification. Further data also is needed for the complementary gene sequences (16S rRNA and COI) for the two investigated specimens. We consequently attribute the split of our two investigated specimens into distinct molecular species to missing data in our analyses.

#### Caecum donmoorei

Taxon classificationAnimaliaLittorinimorphaCaecidae

Mitchell-Tapping, 1979

1D3CEFFF-2817-51DF-AF93-B2ABE7572DAC

Caecum donmoorei Mitchell-Tapping, 1979: 104, 105, figs 21, 22, 31, 32 Type locality: In 5 m of water in Sprat Baz, Water Island, USVI.

##### Material examined.

Panama • 1, juv.; Bocas del Toro; 9.2141, -81.9318; depth 8.5 m; 5 Jun 2010; USNM BRS 2010 exped.; Stat. BRS108; DNA voucher; GenBank: MT704265, MT731691; USNM 1618857. • 1; same collection data as for preceding; DNA voucher; GenBank: MT704266, MT731692; USNM 1618858. Belize • 1 (Fig. 7G); Carrie Bow Cay; 16.8015, -88.0790; depth 10 m; 14 Jan 2010; USNM Belize2010 exped.; Stat. CBC1b; DNA voucher; GenBank: MT704274, MT731703; USNM 1618844.

##### Shell morphology.

Shell opaque white and solid. Size large > 2.0 mm. Tube moderately curved and curvature stronger towards aperture (Fig. 7G). Septum blistered with pointed, sharp mucro (Fig. 7G). Aperture surrounded by three narrow thick rings. Sculpture consists of 25 distinct squarish ribs with wide, deep interspaces. No microsculpture available.

##### Remarks.

The collected specimen USNM 1618844 closely resembles the description of *C. donmoorei* from the Virgin Islands (Mitchell-Tapping 1979), however microsculptural data for comparison is missing. Our molecular data suggests the species identity among specimens with less separated, strongly flattened rings and almost vanishing interspaces (e.g., in juvenile USNM 1618857). The same has been observed for the confusingly similar species *C. quadratum*, Carpenter (1855–1857: 322, pl. 34, fig. 370). It can also exhibit a considerable variety of shell morphologies (Lightfoot 1993a), however it origins from the Eastern Pacific. And a second highly similar species, *C. regulare* (see above) is clearly distinguished herein based on molecular data.

#### Caecum cf. strangulatum

Taxon classificationAnimaliaLittorinimorphaCaecidae

de Folin, 1867

9552E75B-7484-530D-B3A0-CB28BFE8EA47

Caecum strangulatum de Folin, 1867: 82 (with variety *acuta* de Folin, 1867). Type locality: Iles aux Perles, dans la baie de Panama [Pearl Islands, Panama].

##### Material examined.

Panama • 1 juv.; Achotines; 7.6349, -79.9968; depth 10 m; 6 Mar 2016; USNM Achotines2016 exped.; Stat. PA23a; DNA voucher; GenBank: MT727072, MT731727; USNM 1618884. • 1 juv. (Fig. 7O, P, R, T); same collection data as for preceding; ZSM-Mol-20200038. • 1 juv.; Achotines; 7.6207, -80.0013; depth 12 m; 29 Feb 2016; USNM Achotines2016 exped.; Stat. PA14; DNA voucher; GenBank: MT727068, MT704287, MT731721; USNM 1618864.

##### Shell morphology.

Shell fragile, color frosted translucent (Fig. 7O). Shape and size the same as for C. cf. teres juvenile (Fig. 7M, N). Sculpture however appears rough and annular using light microscopy, growth lines more distinct and wavy (Fig. 7T). Septum flat, mucro narrow, finger-like (Fig. 7R). Aperture fringed as typical for a juvenile, growing specimen (Fig. 7P). Striped microsculpture consists of narrow longitudinal and interrupted emarginations, shifted against each other. Almost identical to C. cf. teres (Fig. 7S).

##### Remarks.

We assign the examined specimen to *Caecum strangulatum* (in the juvenile form), which was described from Pacific Panama (de Folin 1867) (holotype MNHN-IM-2000-4586, accessed through the online catalogue of the MNHN), due to the narrow mucro and the annular sculpture, which probably can be interpreted as the transition to a ribbed ornamentation in later life stage. The separate species status of *C. strangulatum* and *C. teres* (see below) is not supported via our molecular species delineation (see Fig. 4). Both are sister clades in phylogenetic analyses (see Fig. 3), but the monophyly of both lineages is not reflected on mitochondrial 16S rRNA (Fig. 4). Both species can be distinguished by morphological features (i.e., a noticeably more pronounced shell structure and a slimmer mucro), but share a unique microsculptural pattern (Fig. 7D). We currently lack comparative data on COI to evaluate whether the results in species delineation on 16S rRNA present a case of incomplete lineage sorting in this recent split between the two sister species or both belong to one genetically and morphologically diverse species. Due to this lack of data, we currently refrain from synonymizing the two yet existing species until more data is available to text species boundaries and the degree of intraspecific variability in shell morphology.

#### Caecum cf. teres

Taxon classificationAnimaliaLittorinimorphaCaecidae

Carpenter, 1857

3E71FF64-B803-5864-80F0-BB5BFD892DD0

Caecum teres Carpenter, 1857: 329, pl. 37, figs 378, 1550. Type locality: Mazatlán [Mexico].

##### Material examined.

Panama • 1 juv.; Achotines; 7.6349, -79.9968; depth 10 m; 6 Mar 2016; USNM Achotines 2016 exped.; Stat. PA23a; DNA voucher; GenBank: MT727070, MT704289, MT731724; USNM 1618865. • 1 juv. (Fig. 7L); same collection data as for preceding; DNA voucher; DNA bank: r462p7f2t91; GenBank: MT704284, MT731718; ZSM-Mol-20200033. • 1 juv.; same collection data as for preceding; DNA voucher; DNA bank: r462p11f2t91; GenBank: MT704286, MT731720; ZSM-Mol-20200037. • 1 juv. (Fig. 7M, N, Q, S); same collection data as for preceding; ZSM-Mol-20200032. • 1 juv.; Achotines; 7.6207, -80.0013; depth 12 m; 29 Feb 2016; USNM Achotines2016 exped.; Stat. PA14; ZSM-Mol-20200027.

##### Shell morphology.

Thin, fragile shell. Color whitish translucent. Length varies from 1.2 to 1.5 mm. Tube elongated, uniformly cylindrical, narrowing towards posterior (Fig. 7M, N). Septum clearly set off from edge of tube with large, triangular mucro with rounded tip (Fig. 7Q). Aperture round with sharp and thin rim. Sculpture appears smooth using light microscopy except for numerous fine horizontal growth lines. Microsculpture composed of numerous narrow longitudinal stripes consisting of serial fine indentations (Fig. 7S). Stripes of indentations are slightly shifted, when intersecting a growth line.

##### Remarks.

Our material from Pacific Panama closely resembles *Caecum teres* (lectotype, NHMUK catalog number 1857.6.4.1550). However, all investigated specimens are juveniles in different growth-stages and identification remains therefore to be confirmed when adult specimens are available for molecular analyses.

#### Caecum

Taxon classificationAnimaliaLittorinimorphaCaecidae

cf . semilaeve Carpenter, 1857

A8F808EC-A6D6-5DC8-98E7-75F5C62C4F1B

Caecum semilaeve Carpenter, 1857: 319, pl. 33, figs 1526. Type locality: Mazatlán [Mexico].

##### Material examined.

Panama • 1 juv.; Achotines; 7.6207, -80.0013; depth 12 m; 29 Feb 2016; USNM Achotines 2016 exped.; Stat. PA14; DNA voucher; DNA bank: r462p2f2t91; GenBank: MT704282, MT731716; ZSM-Mol-20200028. • 1 juv. (Fig. 7U–X); Achotines; 7.6349, -79.9968; depth 10 m; 6 Mar 2016; USNM Achotines 2016 exped.; Stat. PA23a; DNA voucher; DNA bank: r462p8f2t91; GenBank: MT704285, MT731719; ZSM-Mol-20200034.

##### Shell morphology.

Shell very thin, delicate and highly translucent, glossy (Fig. 7U). Size 1.0 mm long, 0.5 mm wide. Tube gradually narrowing towards posterior end and evenly curved. Septum blistered, entirely below posterior tube end (Fig. 7W). Posterior end fringed and in specimen ZSM-Mol-20200034 still connected partly with mucro indicating a recent shedding of transitional septum. Mucro with elongated, rounded tip, only slightly extending from tube (Fig. 7W). Aperture bordered by very tiny sharp lip, otherwise fragile (Fig. 7X). Shell surface smooth, no sculpture visible apart from regular growth lines. Shell covered by organic layer (periostracum).

##### Remarks.

The examined shells all belong to juveniles due to their fragile character and the unfinished aperture. Therefore, it will be critical to reassess these observations based on mature shell structures, as sculpturing is known to be variable during development (see e.g., *C. metamorphosicum* S. Lima, Santos & Absalão, 2013 in Lima et al. 2013). The specimens investigated here build and shed transitional septa as described by Pizzini et al. (1998). Specimens that show a similar mucro are *Caecum lineicinctum* de Folin, 1880 (compare Absalão and Gomes 2001: 10, figs 1, 2), *C. liratocinctum* Carpenter, 1857; however, both occur in the Western Atlantic (de Folin 1880; Moore 1972; Lightfoot 1992a; Absalão and Gomes 2001). *Caecum semilaeve* is a species described as similar to *C. liratocinctum* (Carpenter 1855–1857) and its type locality is Mazatlán, Mexico, Eastern Pacific, thus with geographic proximity to the localities of our investigated specimens (Achotines, Panama, eastern Pacific). We therefore assign our material to *C. semilaeve*. However, identification remains uncertain without having observed the manifestations of the shell sculpture as described for *C. semilaeve* in later developmental stages (compare syntypes C. elongatum var. semilaeve NHMUK 1857.6.4.1526).

## Discussion

### Taxonomic consequences for Caecidae and the fate of *Meioceras*

The Caecidae are currently classified in ten genera (MolluscaBase 2019) of which two, *Caecum* and *Meioceras*, can be found in the Central American region. We investigated two species classified as *Meioceras* and 15 *Caecum* species, including one species new to science (*C. invisibile* sp. nov.), and two candidate species (MOTU I and II). Three individuals that were originally assigned to *Meioceras* are resolved among species of the genus *Caecum* in our molecular phylogenetic analyses and, moreover, have independent evolutionary origins within *Caecum* (Fig. 3). Our data confirms the existence of at least two valid *Meioceras* species (i.e., *M. nitidum* and *M. cubitatum*), which however, based on our data should be reassigned genus *Caecum*. For taxonomic stability, we refrain to reallocate these species at present, until the molecular sampling can be expanded and further data is available supporting our initial results. Unfortunately, we lack material of *M. cornucopiae* (the type species by subsequent designation) and of a putative fourth species, *M. tumidissimum*, both described from Brazil (de Folin and Périer 1869). These taxa are needed to settle the debate on the number of species and to clarify the validity of the genus *Meioceras*. Our findings indicate, however, that the more bulbous shell in *Meioceras* when compared to a more tube-like shell in *Caecum* might not justify generic subdivision. Moreover, the diagnostic spiral growth pattern in the larval and juvenile shell of *Meioceras* (Bandel 1996) might not be used for unambiguous discrimination as our phylogeny indicates that such patterns have evolved independently at least two times within the genus *Caecum*. The bulbous adult shell with an oblique constricted aperture was thought to develop from the preceding ontogenesis of a helicoidal shell section, observable at the beginning of the second growth stages (De Folin 1880; Absalão and Pizzini 2002). This diagnosis has been problematic because in the past other species (Absalão and Pizzini 2002) that also express a similar shell-shape had been classified as *Caecum* due to a lack of observations of the juvenile stadia or the lack of the aforementioned growth pattern (e.g., *Caecum ryssotitum*, Bandel 1996). By contrast, species with typical tube-shaped *Caecum*-shells are known to also have curved growth axes (e.g., *C. antillarum* and *C. japonicum* referred to as *C. glabellum* in Bandel 1996: 65, figs 8, 9). Differences between these growth patterns and those of *Meioceras* seem to be negligible. Thus, it remains to be tested whether the remaining two *Meioceras* species form a monophylum separate form *Caecum* and whether alternative diagnostic morphological or molecular characters can be found to justify the generic subdivision of Western Atlantic Caecidae. Alternatively, such studies may confirm that *Meioceras* is a junior synonym of *Caecum*. The present study might also have consequences for the recently established genus *Mauroceras*, which unites Indo-Pacific Caecidae formerly classified as *Meioceras* (Vannozzi 2019). But, in contrast to *Caecum* and Western Atlantic *Meioceras*, which cannot be clearly separated based on variable growth patterns, *Mauroceras* is diagnosed by a planorbid protoconch with a clear sinusigera, which at present justifies its generic status.

### Phylogenetic interpretation of shell morphologies and general insights for shell-based taxonomy

The taxonomy of Central American Caecidae has been based on macroscopic shell characters and, consequently, type-species are often poorly defined, and has made the established taxonomy prone to multiple descriptions of synonyms and the establishment of ambiguous species-complexes that are typical for many clades of micromolluscs (Golding 2014b). Modern microsculptural analyses have greatly increased the reliability of shell-based taxonomy and the availability of diagnostic characters in the otherwise largely featureless caecid shells (Pizzini et al. 2013; Vannozzi 2017). However, distinct shells based on coarser diagnostic features can have a similar microsculpture (Vannozzi 2017), suggesting that shell microsculpture should be co-evaluated with traditional diagnostic features and, indeed, that it might be especially valuable to discriminate closely related species. In Central American Caecidae the presence of a series of morphologically highly-similar ribbed taxa (i.e., *C. compactum* Carpenter, 1857, *C. quadratum*, *C. clathratum*, *C. gurgulio* Carpenter, 1858, *C. pulchellum*, *C. regulare*, *Caecum planum* de Folin, 1874) with controversial species status and inconsistent synonymization (Moore 1972) are especially problematic. Here we report SEM-based shell microsculpture that can distinguish taxa and justify the independent species status of *C. regulare* and *C. pulchellum* (compare fusiform lobes (Fig. 7C) with fine longitudinal lamellae (Fig. 7A). However, *C. clathratum* does not possess a unique microsculpture and we are lacking SEM data for our investigated specimen of *C. donmoorei*. Nevertheless, molecular species delineation analyses confirm the existence of four genetically distinct species among those Central American ribbed caecids (i.e., *C. pulchellum*, *C. regulare*, *C. donmoorei* and C. cf. clathratum), highlighting the value of complementary molecular analyses to detect possible synonyms or confirm the validity of existing species in taxonomically problematic species complexes.

The different growth stages of caecid development present an additional problem for taxonomic circumscription, which cannot be overcome easily by microsculptural analyses because, the shape and some patterns of ornamentation appear late in development. This often results in the incorrect assignment of different growth stages even at the generic level (Absalão and Pizzini 2002). In consequence, it requires time consuming comparisons of hundreds of shells for reliable species description and identification (Lightfoot 1992a, 1993a), unfeasible in modern times of taxonomic impediment. The molecular analyses presented in this study show that barcoding markers (i.e., partial mitochondrial COI and 16S rRNA genes) are a valuable tool to address the challenges of caecid taxonomy and that molecular species delineation analyses can reliably identify groups of closely related specimens, therewith providing objective data on intraspecific variability of shell characters. Above all, they enable an unambiguous assignment of juvenile forms in different growth stages to their fully developed adult morphologies (see e.g., *C. heptagonum* in Fig. 5G, *C. pulchellum* and C. cf. teres in Fig. 7A, D). Based on a purely morphological approach, these juveniles would have remained unidentified and unaccounted for in biodiversity data, and their contribution to caecid diversity would have been lost. However, in some cases, juveniles could not be matched to their adult counterparts using molecular data since we had no adult animals in our sample. These taxa identified by the molecular data could not be named (e.g., *Caecum* sp. MOTU II). These examples highlight, how the successful identification of juveniles lacking morphological diagnostic features by means of their genetic fingerprints requires an extensive barcode library of Central American Caecidae as a taxonomic reference. The barcodes of the morphospecies investigated here are the first contribution to such a reference library that can help to provide a baseline and enhance future identification. In general, the poor taxonomic coverage of gastropods and marine invertebrates in public molecular databases such as NCBI GenBank has been identified as a major obstacle to making effective use of molecular barcoding approaches (e.g., to assign spawn to adult specimens; Puillandre et al. 2009). Thus, it is hoped that in the future the scientific community will be able to invest more of its financial and personnel capacities in integrative faunistic approaches that strengthen fundamental biodiversity research.

In biodiversity assessment and conservation biology, molecular species delineation has also demonstrated its potential for identifying cryptic species (Bickford et al. 2007; Jörger et al. 2012; Lemer et al. 2014; Leasi et al. 2016). In revealing cryptic taxa, our study indicates that the species diversity of caecids may have been underestimated until now. Unsurprisingly, the cryptic species, which we identified, are those of particularly small, feature-poor, caecids with few diagnostic characters (see Fig. 5C–F). Indeed, our analyses suggest that meiofaunal character-poor caecids (assigned to the ‘*Caecum glabrum*-like’ species complex) have evolved several times independently from the larger ornamented caecids in the Central American region. The same may have happened in the northern Atlantic *C. glabrum* and Northwest Pacific *C. glabellum* Adams, 1868 from Japan. The evolution of a tubular shell marks the origin of Caecidae and likely correlates with a transition to an infaunal lifestyle (e.g., among corals and coral rubble or algae; Bandel 1996). However, interstitial habitats are very variable, differing with regards to the available space between the sand grains which influences the mobility, light intensity and therefore visibility and protection from predators. In the ‘*Caecum glabrum*-like’ microsnails, the morphological similarity among taxa (i.e., minute, slim shell, lack of ornamentation and coloration) likely correlates with a habitat shift into the mesopsammon and the consequent habitat restrictions of this special interstitial environment. ‘Regressive evolution’ leading to simplified and highly adapted body plans are typical for the mesopsammon (Swedmark 1968) and consequently the associated meiofauna is prone to cryptic speciation (Jörger et al. 2012; Meyer-Wachsmuth et al. 2014; Leasi et al. 2016).

## Conclusions

Our study of Central American Caecidae shows that traditional taxonomic shell characters cannot sufficiently describe the diversity of these microsnails. Microsculptural investigations add valuable additional information for correct taxonomic assignment, species delineation, and the evaluation of gross shell morphological variation within and among species. However, its effectiveness in allocating juvenile growth stages or morphologically rather cryptic species with few diagnostic shell characters into the classificatory system remains limited. This limitation in morphology-based approaches was overcome by integrating genetic barcoding data and molecular species delineation which revealed a complex of cryptic lineages that were potentially associated with a habitat shift from an epibenthic to (temporary) mesopsammic lifestyle among the interstices of sand grains and shell hash. Integrative biodiversity assessments help contribute to a barcoding library of genetic fingerprints of the targeted fauna which enable rapid identification of new samples and is linked to the existing taxonomic history by morphological identification of the voucher specimens. Thus, beyond documenting the shell in microstructural detail, whenever possible a shell voucher should remain intact available for future investigation when novel methods approach. Nevertheless, the vast accumulation of potential synonyms and old names in gastropod taxonomy is problematic, and species need to be taxonomically revised prior to establishing names for newly discovered species. Re-collecting at type localities might not always be feasible for each species, especially when revising large groups with many described species. Additionally, it bears the risk of false identification when cryptic species co-occur at small geographical ranges. However, genetic barcodes have been generated successfully from old mollusk samples in natural history collections – wet material (Jaksch et al. 2016) and dried shells (Der Sarkissian et al. 2017) alike – and hopefully advances in genetic methodology will soon provide cost-efficient and reliable workflows to also adapt them to microsnails as a complement towards ongoing biodiversity studies.

## Supplementary Material

XML Treatment for Meioceras

XML Treatment for Meioceras nitidum

XML Treatment for Meioceras cubitatum

XML Treatment for Caecum

XML Treatment for Caecum cf. corrugulatum

XML Treatment for Caecum invisibile

XML Treatment for Caecum heptagonum

XML Treatment for Caecum imbricatum

XML Treatment for Caecum cooperi

XML Treatment for Caecum debile

XML Treatment for Caecum striatum

XML Treatment for Caecum cf. clathratum

XML Treatment for Caecum pulchellum

XML Treatment for Caecum regulare

XML Treatment for Caecum donmoorei

XML Treatment for Caecum cf. strangulatum

XML Treatment for Caecum cf. teres

XML Treatment for Caecum
